# Transcriptional profiles analysis of effects of *Toxoplasma gondii* rhoptry protein 16 on THP-1 macrophages

**DOI:** 10.3389/fcimb.2024.1436712

**Published:** 2025-01-28

**Authors:** Ningai Yang, Mingyang Li, Hong Yang, Jiaming Li, Tiantian Dang, Guangqi Li, Zhijun Zhao

**Affiliations:** ^1^ Institute of Medical Sciences, General Hospital of Ningxia Medical University, Yinchuan, Ningxia, China; ^2^ Diagnosis and Treatment Engineering Technology Research Center of Nervous System Diseases of Ningxia, Yinchuan, Ningxia, China; ^3^ Ningxia Key Laboratory of Clinical Pathogenic Microorganisms, Yinchuan, Ningxia, China; ^4^ Department of Cardiology, Cardiovascular and Cerebrovascular Disease Hospital, General Hospital of Ningxia Medical University, Yinchuan, Ningxia, China; ^5^ Ningxia Hui Autonomous Region Hospital of Traditional Chinese Medicine and Research Institute of Traditional Chinese Medicine, Yinchuan, Ningxia, China; ^6^ Medical Laboratory Center, General Hospital of Ningxia Medical University, Yinchuan, Ningxia, China; ^7^ Ningxia Medical Laboratory Clinical Research Centre, Yinchuan, Ningxia, China

**Keywords:** *Toxoplasma gondii*, ROP16, RNA-seq, THP-1, macrophage

## Abstract

**Introduction:**

*Toxoplasma gondii*, an intracellular parasitic protozoan, is globally recognized for its ability to cause parasitic diseases and has developed diverse strategies to evade immune-mediated elimination. The protein ROP16 of *T.gondii* plays a crucial role in this evasion process by specifically targeting macrophages and mononuclear phagocytes *in vivo*. However, the precise mechanisms underlying the involvement of type II ROP16 proteins in infection, inflammation, and other processes remain unknown.

**Methods:**

To investigate the mechanism of action of gonococcal ROP16 proteins in human macrophages, we constructed a lentivirus overexpressing ROP16 and established stably transfected cell lines. We then analyzed the gene transcriptional profiles of ROP16 II in THP-1 macrophages using transcriptome sequencing. Interaction networks were constructed by screening differentially expressed genes and performing gene function enrichment analysis.

**Results:**

As a result, five differentially expressed genes were identified: AAMDC, GPR158, RAD9A, STOML1, and STRA13. Immuno-featured differential analysis showed that type 17 T helper cells were more strongly correlated with GPR158 and STRA13, while CD8 T-cell was most strongly correlated with STOML1.

**Discussion:**

Therefore, we conclude that the ROP16 protein plays a pivotal role in THP-1 macrophage infection and these five differentially expressed genes may serve as promising molecular targets for the prevention or control of toxoplasmosis. These findings have significant implications for the diagnosis and treatment of toxoplasmosis.

## 1 Introduction

*Toxoplasma gondii*, a member of the phylum Parietoidea, class Sporozoa, order Eukarya, family Isosporoidea and genus Toxoplasma, is an opportunistic pathogenic protozoan (David Sibley et al., 2009). It is a parasitic organism capable of causing zoonotic diseases and can intracellularly infect the cells of all eukaryotic organisms except erythrocytes. *T.gondii* can invade the nucleated cells of various warm-blooded animals, including humans, and is responsible for global parasitic diseases. While most *T.gondii* infections are asymptomatic or mild, clinical manifestations primarily occur in immunodeficient or immunocompromised individuals such as pregnant women or those with HIV infection (Sun et al., 2021).

ROP16, a rhoptry protein of *T.gondii*, exhibits serine-threonine kinase activity and serves as an essential virulence factor during host cell invasion (Young et al., 2020). The pivotal role of ROP16 in evading the immune response of the host has been extensively demonstrated (Chang et al., 2015). Notably, studies have revealed that ROP 16 I/III (from type I and type III strains) phosphorylates Stat6/Stat3, inducing early polarization towards alternatively activated macrophages (M2) upon infection (Wang et al., 2018). Furthermore, compared to type I parasites, infection with type II parasites leads to up-regulation of IL-12 p40 production in macrophages (Yamamoto et al., 2009).

Relevant studies have demonstrated that transfection of Toxoplasma rhoptry protein 16 in SH-SY5Y human neuroblastoma cells induces alterations in the host cell’s transcriptional profile, affecting the expression of multiple genes. Notably, these genes play crucial roles in nervous system development, apoptosis and transcriptional regulation. Their dysregulation may contribute to both the host cells response against *T.gondii* infection and the pathogenesis of Toxoplasma (Fan et al., 2016). Additionally, Hengming Ye et al. revealed that *Toxoplasma gondii* suppresses the proliferation and migration of breast cancer cells by modulating their transcriptome (Ye et al., 2024).Although *T.gondii* can infect various types of nucleated cells, macrophages and associated mononuclear phagocytes are its preferred targets *in vivo*. The parasite has multiple ways to evade immune-mediated killing (Li et al., 2017). Immune cells, including natural killer cells, mast cells and helper T cells, also contribute to the immune response during toxoplasma infection. Conventional natural killer cells are critical for early immunity to against *T. gondii* infection (Ivanova et al., 2016). Studies have shown that Toxoplasma WH3Δ *rop16* strain impairs the function of T regulatory cells (Tregs) (Wang et al., 2018). Mast cells play an important role in immunity against certain infections, as well as in allergy, and inflammation (Ekoff et al., 2007). Therefore, studies targeting macrophages are crucial for understanding the underlying strategies of parasite-host interactions. In recent years, there have been many national and international studies focusing on using *T.gondii* to infect human macrophages (Murray, 2011). Macrophages are important defense cells that protect body against invasion by foreign pathogens and serve as ideal cellular models for studying the mechanisms associated with *T.gondii* infection (Shapouri-Moghaddam et al., 2018). However, there are relatively few studies examining the role of the ROP16 protein in human macrophages and some suggest that different types of ROP16 can polarize macrophages into classically activated M1 or alternatively activated M2 phenotypes (Hu et al., 2022). Therefore, the specific molecular mechanism of Toxoplasma ROP16 protein in macrophages remains unclear. In this study, we successfully constructed stable cell lines overexpressing ROP16 type II protein and performed transcriptome analysis to better understand its mechanism in THP-1 macrophages.

## 2 Materials and methods

### 2.1 Construction of lentiviral vector overexpressing *rop16* gene and establishment of stable cell line

**Figure 1:**
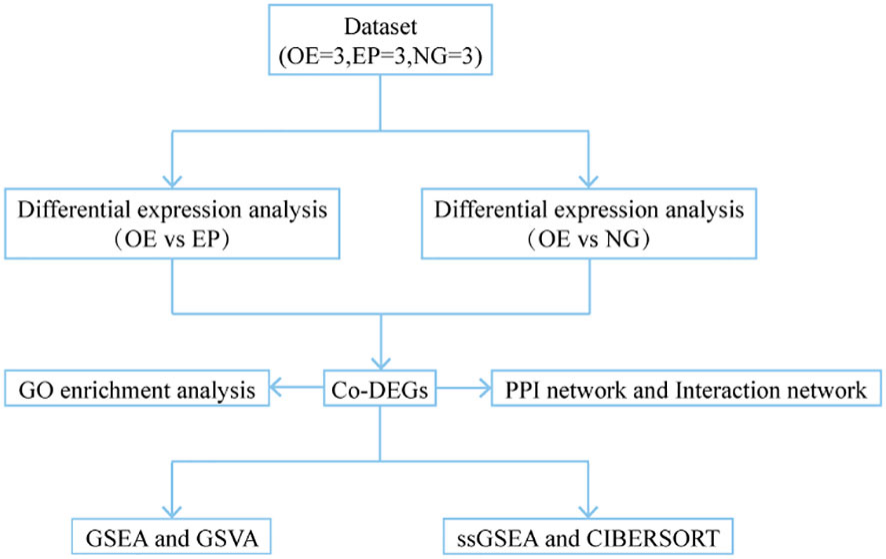
Technology Roadmap. OE, Overexpression group; EP, Empty group; NG, Normal group; Co-DEGs, Common differentially expressed genes; GO, Gene Ontology; GSEA, Gene Set Enrichment Analysis; GSVA, Gene Set Variation Analysis; ssGSEA, single-sample gene-set enrichment analysis.

The total RNA of ME49 strain was extracted using the Trizol method, and the purity and concentration of the total RNA were assessed, and the cDNA of ME49 strain was obtained by reverse transcription using a reverse transcription kit. Based on the *rop16* gene sequence, the following primers were designed: forward 5 ‘- GCGAATTCACCATAGAAAGTGACCACGAAAGGGCTTGC - 3’, and reverse 5’-GATCAGCGGCCTACATCCGATGTAAAGAAAGTTCGGTAGTTG-3’. Primers containing his-tag were designed to amplify the *rop16* gene fragment. The resulting PCR fragment and pCDH-MSCV empty vector were double-digested using EcoR-I and Not-I enzymes after PCR amplification, converting blunt ends into sticky ends for ligation. The PCR fragment and vector were ligated using T4 ligase and transformed into DH5αcompetent cells, from which positive clones of the transformants were selected for sequencing and identification. The positive clones were selected for sequencing and identification. Human embryonic kidney cell line 293T was transfected with *rop16* lentiviral vector packaged in a triple plasmid system (pMAL +VsVG +pREV). The supernatant was collected, concentrated and purified, and viral titer was determined. Three groups were established: the *rop16* Overexpression Group (OE), an pCDH-MSCV Empty vector Group(EP) and a blank control Normal cell Group(NG) (**Figure 1**). In 6-well plates, pCDH-MSCV-ROP16 overexpression virus solution, pCDH empty vector virus solution and culture medium were added respectively. THP-1 macrophages induced by Phorbol-12-myristate-13-acetate (PMA) were then infected, and stable cell lines overexpressing *rop16* were generated through puromycin selection, and the expression of GFP was observed using fluorescence microscope. Western blotting and RT-qPCR were used to detect the protein levels and mRNA expression of *rop16*, respectively.

### 2.2 Data processing and analysis of differential representation

We utilized R’s limma package (David Sibley et al., 2009) to compare samples from the Overexpression group (OE) with those from the Normal group (NG) and the Empty group (EP). Differentially expressed genes (DEGs) were identified between the groups using a significance threshold of *P*< 0.01. Subsequently, we determined the intersection of DEGs between the two comparisons. The co-differentially expressed genes (Co-DEGs) were identified, representing the genes with common differential expression between the OE and NG groups, as well as between the OE and EP groups. A t-test was employed to further screen for differentially expressed genes between the OE and NG groups and between the OE and EP groups. Applying the same significance criterion of *P*< 0.01, we again identified the intersection of DEGs across the different comparisons to derive the Co-DEGs. Finally, the Co-DEGs obtained from the limma package differential analysis were intersected with those derived from the t-test, resulting in a final set of Co-DEGs for subsequent analysis.

### 2.3 Gene functional enrichment analysis

Gene Ontology (GO) (Murray, 2011) analysis encompasses three main categories: biological process (BP), molecular function (MF) and cell component (CC). GO enrichment analysis of Co-DEGs was performed by using the R software package clusterProfiler, with a selection criterion of *P*<0.05. The *P* correction method was Benjamini-Hochberg (BH) (Gan et al., 2017).

### 2.4 Construct PPI interaction network

The GeneMANIA (Li et al., 2021) database was utilized to generate hypotheses regarding gene function, analyze gene lists, and prioritize gene function analyses. It can assign weights to each functional genome dataset based on the predicted value of the query and make gene function predictions. Given a query gene, GeneMANIA identifies genes that are likely to share functions with it, based on their interactions. Genes with similar functions to Co-DEGs were predicted through GeneMANIA online platform, and the interaction network was downloaded. In the figure, the inner circle represents the Co-DEGs, the outer circle depicts the genes with similar functions, and the line colors correspond to the line represents the interconnected functions in our study.

### 2.5 Construct the interaction network of mRNA-RBP, mRNA-miRNA, mRNA-TF and mRNA-drug

The ENCORI database analyzes microRNAs-mRNA interactions through data mining, which provides multiple visual interfaces for exploring miRNA targets. We used ENCORI (Mosser and Edwards, 2008) interacted with Co-DEGs (RNA binding proteins). clipExpNum > =5 to screen mRNA-RBP interactions and map interaction networks.

We used ENCORI database to predict the mirnas interacting with Co-DEGs, and also screened the mirnas interacting with pancancerNum>=5, and mapping the interaction.

The CHIPBase database predicts transcriptional regulatory relationships between many genes and transcription factors (TFS). We searched for Co-DEGs binding transcription factors (TFS) in the CHIPBase database by the number of samples found (upstream) >0 and number of samples found (downstream)>0 was used as a screening criterion to screen interaction relationships and visualize mRNA-miRNA interaction networks via Cytoscape software.

Potential drugs or small molecule compounds that interact with Co-DEGs are predicted using the public Comparative Toxicology Genomics Database (CTD), with a “reference count” > 0 was used as a screening criterion to screen MRNA-drug interaction pairs. Cytoscape software is used to visualize mRNA-drug interaction networks.

### 2.6 GSEA enrichment analysis between OE group and NG group

Gene Set Enrichment Analysis (GSEA) (Kong et al., 2015) is commonly used to analyze changes in the activity of pathways and biological processes in samples. Based on the results of the previous differential analysis between the overexpression and normal group (OE/NG), we divided all genes into logFC value related positive and negative groups according to the positive and negative order of logFC value of genes between the two groups. Gene enrichment analysis was performed using clusterProfiler software package. The specific parameters are as follows: the seed is 2022, the number of calculations is 1000, the number of genes in each gene set is at least 10, the number of genes in each gene set is at most 500, and the *P*-value correction method is Benjamini-Hochberg (BH). We from Molecular Signatures Database (MSigDB) (Croken et al., 2014) Database access “c2. Cp. All. V2022.1. Hs. Symbols. The GMT [all Canonical Pathways] (3050)” gene set, The screening criteria for significant enrichment were p. Adj < 0.05 and FDR value (q.value) < 0.05.

### 2.7 GSVA between OE group and NG group

Gene Set Variation Analysis (GSVA) (Deichaite et al., 2022) evaluates the genomic enrichment of the transcriptome on a chip by converting the gene expression matrix between different samples into an inter-sample gene expression matrix. To assess whether different pathways are enriched in different samples. We performed “GMT “GSVA analysis of the gene expression matrix of the overexpressed and normal groups through the MSigDB (Molecular Signature Database) database to calculate the functional differences of the enrichment pathway between the two groups. In this study, from the pathways with *P*<0.05, 10 pathways each with the largest and smallest logFC were selected for subsequent analyses.

### 2.8 Identification of immune infiltrating cells and correlation analysis

A single sample genomic enrichment analysis (ssGSEA) algorithm was used to quantify the relative abundance of different immune cell infiltrations, while labeling the infiltrated immune cell types, such as CD8+ T cells, and various human immune cell subtypes, such as denatured cells, macrophages, and regulatory T cells. We obtained the enrichment scores of each immune cell by analyzing and calculating the ssGSEA algorithm in the GSVA (Li et al., 2022) package of R package (version 1.46.0), and the relative abundance of each type of immune cell infiltration in each sample is represented by the enrichment fraction. The difference of immune cell infiltration abundance between OE group and NG group was shown by block diagram. The correlation between immune cells is shown by a lollipop chart. The correlation between immune cells and Co-DEGs was visualized by the correlation dot plot drawn by the R package ggplot2.

CIBERSORT (Bao et al., 2020) is an analytical algorithm for estimating the composition and abundance of immune cells in mixed cell populations. We screened immune cells with enrichment scores greater than 0 by CIBERSORT. The correlation between immune cells was calculated using spearman statistical algorithm and visualized using R software package ggplot2. The difference in the abundance of immune cell infiltration between the OE group and the NG group was shown by the columnar stack diagram. The correlation between immune cells and Co-DEGs was calculated by spearman statistical algorithm, and the correlation point plot was drawn by R software package ggplot2.

### 2.9 Quantitative real-time PCR for differentially expressed genes

Total RNA was extracted from the cells of NG, EP and OE groups by Trizol method, reverse transcribed into cDNA, and the mRNA levels of AAMDC, GPR158, RAD9A, STOML1, and STRA13 genes were determined by RT-PCR in the cells of different groups. The results were detected by Light Cycler 480 system PCR instrument software and analyzed to obtain the Ct values, and the relative expression of mRNA of cell-related factors in the three groups was calculated by applying the 2-△△Ct value, The mRNA expression of the cells in the NG group was set to 1, and all experiments were performed in 3 replications. Differential gene primer sequence are as follows: AAMDC, 5 ‘-TTGGCCGAGGGATGAGTGA-3 ‘ and 5’-GCAACCAAGGCATTATACTCCT-3 ‘; GPR158, 5 ‘-ATCTACGGGTTGCAGCCTAAC-3 ‘ and 5’-AACCAGCCATCACTTGAGCAT-3 ‘; RAD9A, 5 ‘-CATTGACTCTTACATGATCGCCA-3 ‘ and 5’-GCCAGGTGAAAGGGAAATGG-3 ‘; STOML1, 5 ‘-GGGAGCCGATGTCCAGTTTC-3 ‘, and 5’-CTGGTCGCTGATCTTGAGC-3 ‘; STRA13, 5 ‘-ATCCAGCGGACTTTCGCTC-3 ‘ and 5’-TAATTGCGCCGATCCTTTCTC-3 ‘.

### 2.10 Statistical analysis

All data processing and analysis in this paper are based on R software. For the comparison of continuous variables between the two groups, an independent student t test was used to estimate the statistical significance of the normally distributed variables. The differences between the non-normally distributed variables were analyzed by Mann-Whitney U test (Wilcoxon rank sum test). If not otherwise specified, the results are calculated using the Mauman correlation analysis method. All statistical *P*-values were bilateral, with a *P*-value less than 0.05 indicating statistical significance.

## 3 Results

### 3.1 To verify the expression of ROP16 in THP-1 macrophages

**Figure 2:**
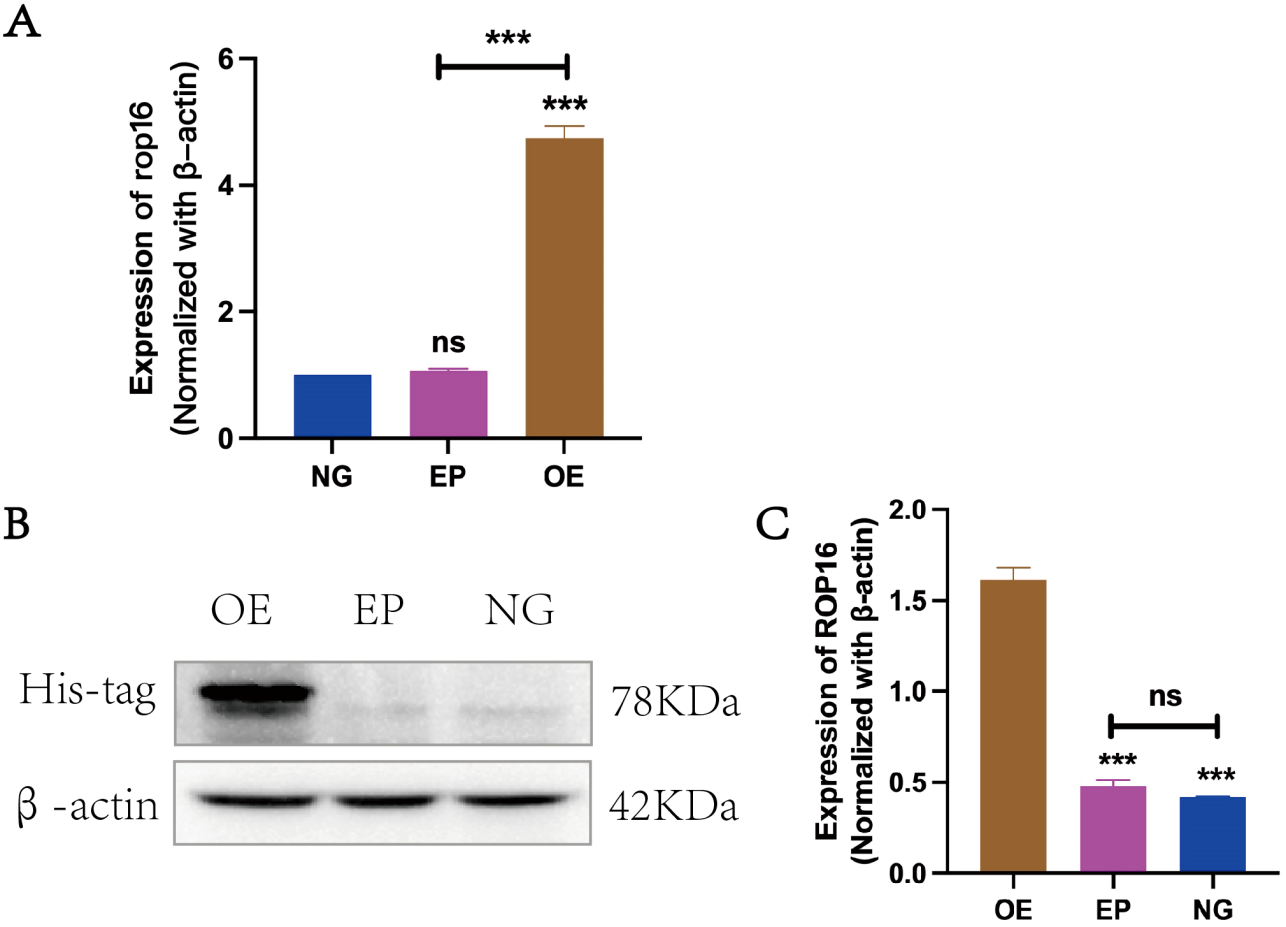
Expression of ROP16 in THP-1 Macrophages: **(A)**. RT-PCR to detect the expression of *rop16* mRNA in THP-1 macrophages of ZC(normal growth), EP(Empty growth) and OE(overexpression) groups after transfection with pCDH-ROP16. **(B, C)**. Blot was used to detect ROP 16 in cells of ZC, EP and OE groups for gray-scale analysis. The ROP 16 protein was detected by His-tag antibody. The internal parameter uses β-actin.The symbol ns was equivalent to *P* ≥ 0.05, which was not statistically significant. The symbol *** is equivalent to *P* < 0.001 and highly statistically significant. OE, Overexpression group; EP, Empty group; NG, Normal group.

In this study, β-actin was employed as an internal reference for RT-qPCR and western blot analysis to compare the transcriptional levels of ROP 16 among the overexpressed group (OE), empty carrier group (EP) and normal control group (NG). The findings demonstrated successful transfection of ROP 16 into THP-1 macrophages (**Figure 2**).

### 3.2 Screening differentially expressed genes

**Figure 3:**
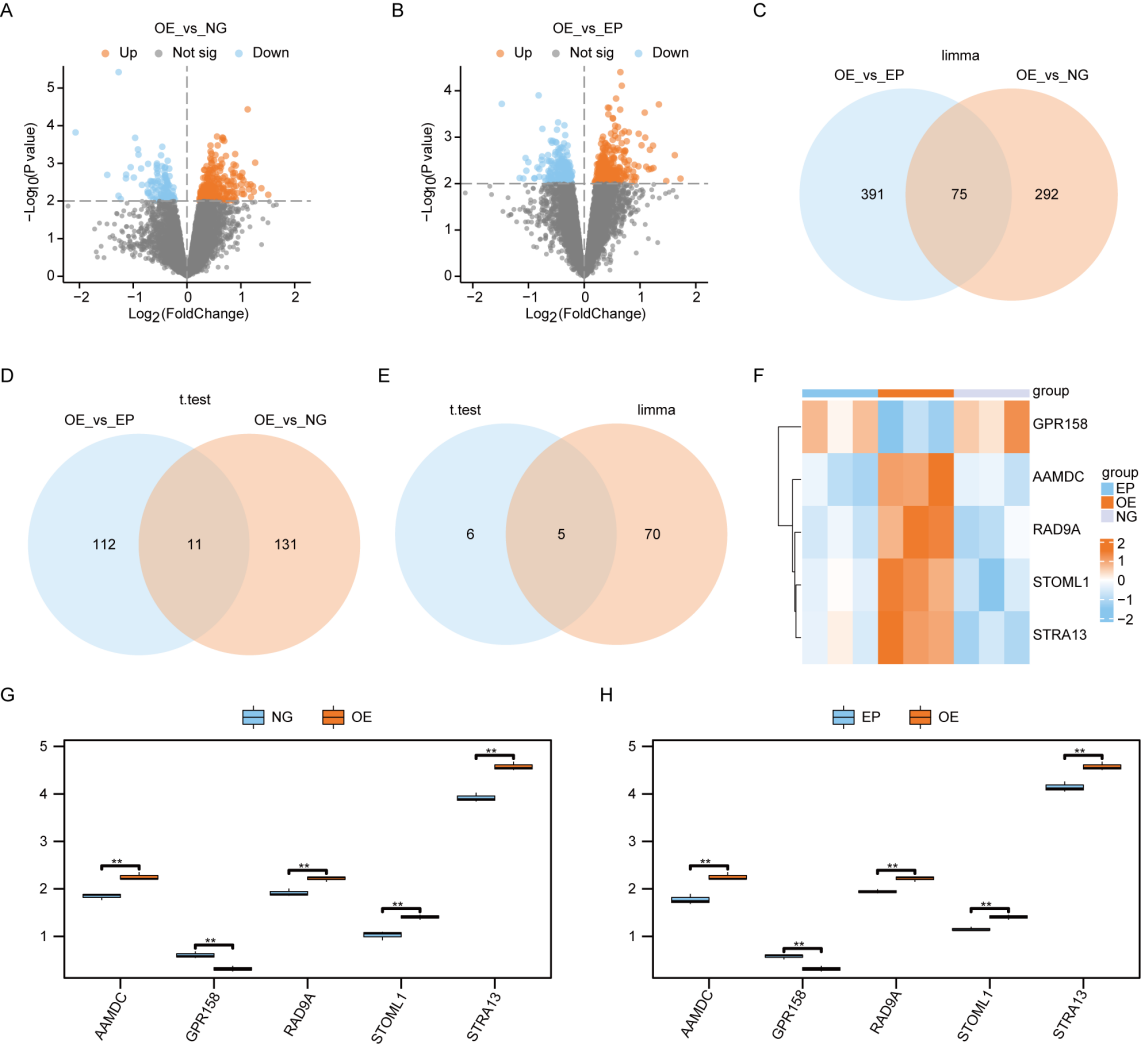
Screening of differentially expressed genes. **(A)**. Volcano plot of the results of limma package difference analysis between OE and NG groups. **(B)**. Volcano plot of limma package difference analysis results between OE and EP groups. **(C)**. Venn diagram of the results of limma packet difference analysis between OE and EP groups, and the results of limma packet difference analysis between OE and NG groups. **(D)**.Venn diagram of the results of t-test difference analysis between OE and EP groups, and the results of t-test difference analysis between OE and NG groups. Venn diagram of Co-DEGs obtained by difference analysis of **(E)**. lomma package and Co-DEGs obtained by difference analysis of t test. Heat map of **(F)**.Co-DEGs expression among the three groups. Group comparison map of Co-DEGs between G-H.O and NG groups **(G)** and OE and EP groups **(H)**. The symbol ns was equivalent to *P* ≥ 0.05, which was not statistically significant. The symbol ** is equivalent to *P* < 0.01, which is highly statistically significant and highly statistically significant. OE, Overexpression group; EP, Empty group; NG, Normal group; Co-DEGs, Common differentially expressed genes.

We performed a differential analysis of all genes between the Overexpression group (OE) and Normal group (NG) samples, as well as between the OE and Empty group (EP) samples, using the limma package in R. The significance threshold was set at *P*< 0.01. A total of 367 differentially expressed genes were identified between the OE and NG groups, with 260 genes showing higher expression in the OE group compared to the NG group, and 107 genes showing lower expression in the OE group compared to the NG group. Similarly, we found a total of 466 differentially expressed genes between the OE and EP groups, with 289 genes exhibiting higher expression in the OE group relative to NG, and 177 genes displaying lower expression in the OE group relative to NG. The results of differential analysis were visualized using volcano plot (**Figures 3A, B**). Subsequently, we conducted an intersection analysis on these two sets of differentially expressed genes to identify a subset of 75 co-differentially expressed genes (Co-DEGs) (**Figure 3C**).

We employed t-tests to identify differentially expressed genes between the OE group and NG group, as well as between the OE group and EP group. Using a significance threshold of *P*< 0.01, we identified 142 differentially expressed genes between the OE and NG groups, along with 123 differentially expressed genes between the OE group and EP group. The overlapping set of differentially expressed genes yielded 11 co-expressed differentially regulated genes Co-DEGs (**Figure 3D**).

The Co-DEGs obtained through differential analysis using the limma package and t-test were intersected (**Figure 3E**), resulting in the identification of five final Co-DEGs: AAMDC, GPR158, RAD9A, STOML1, and STRA13.

We subsequently generated an expression heat map to show the differential expression patterns of 5 Co-DEGs across the three experimental groups (**Figure 3F**). This analysis revealed a significant divergence in the expression profiles of these genes between the OE group and the other two groups.

We generated group comparison maps for 5 Co-DEGs between the NG and OE group (**Figure 3G**), as well as between the EP and OE group (**Figure 3H**). The results revealed significant differences in the expression of these 5 Co-DEGs between the NG and OE groups (**Figure 3G**), as well as between the EP and OE groups (**Figure 3H**).

### 3.3 Functional enrichment analysis of Co-DEGs

**Figure 4:**
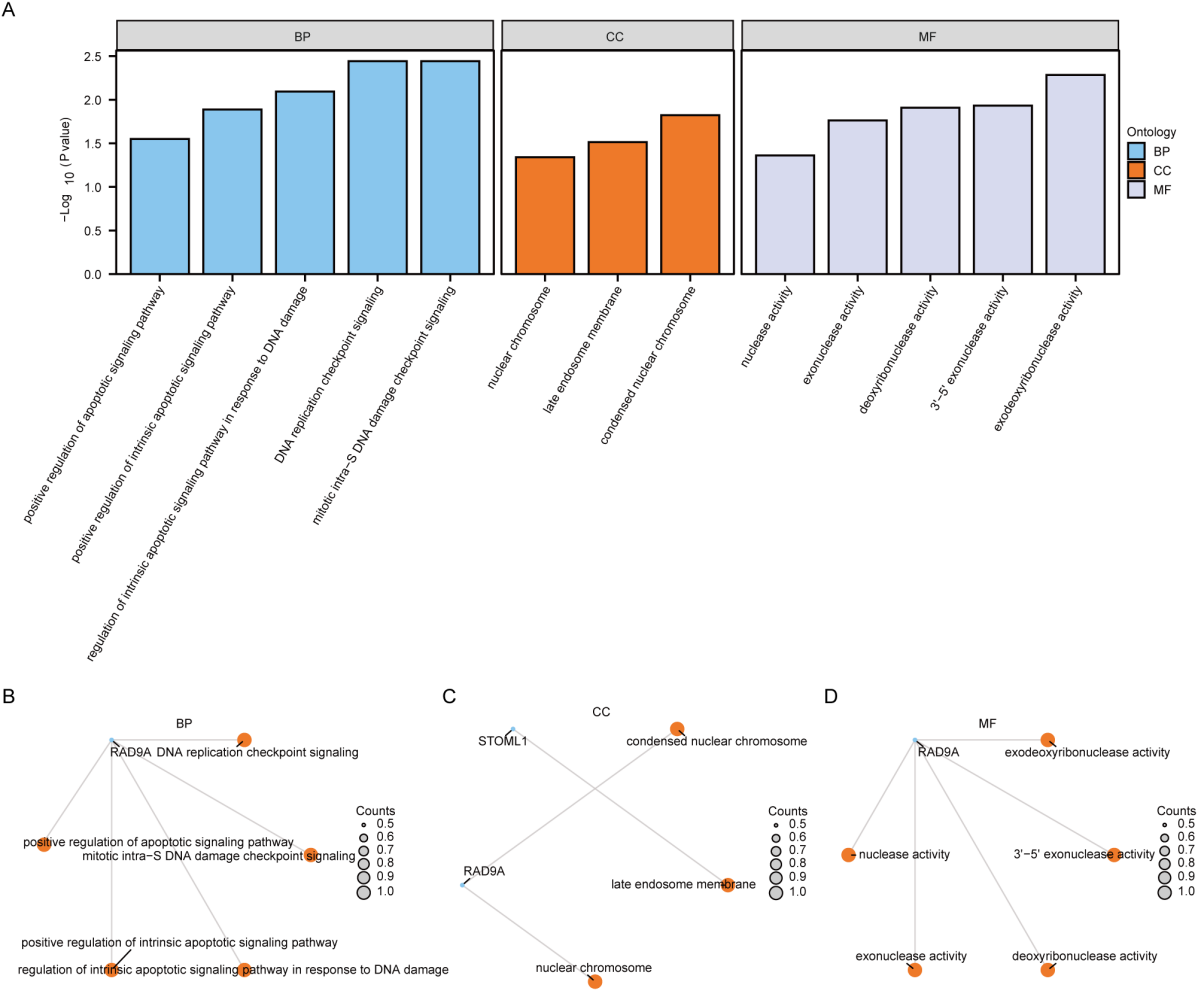
Functional enrichment analysis (GO) of Co-DEGs. The bar chart of GO enrichment analysis results of **(A)** co-degs is displayed. B-d. Circular network diagram of BP pathway **(B)**, CC pathway **(C)**, and MF pathway **(D)** in GO functional enrichment analysis results of Co-DEGs. In the bar graph **(A)**, the abscissa is the GO terms s, and the height of the bar represents the *P* value of the GO terms or KEGG terms. In the network diagram **(B-D)**, blue dots represent specific genes, and orange dots represent specific pathways. Co-DEGs, Common differentially expressed genes; GO, Gene Ontology; BP, biological process; CC, cellular component; MF, molecular function; The screening criterion for GO enrichment items was *P*< 0.05.

To investigate the relationship among biological processes, molecular functions, cellular components and biological pathways of 5 Co-DEGs (AAMDC, GPR158, RAD9A, STOML1, STRA13), we conducted Gene Ontology (GO) gene function enrichment analysis for these genes. Enriched items were screened based on a significance level of *P*< 0.05, and pathways meeting the screening criteria were considered statistically significant. The results revealed that the 5 Co-DEGs were primarily enriched in DNA replication checkpoint signaling, mitotic intra-S DNA damage checkpoint signaling, mitotic intra-S DNA damage checkpoint signaling, regulation of intrinsic apoptotic signaling pathway in response to DNA damage, positive regulation of intrinsic apoptotic signaling pathway, positive regulation of apoptotic signaling pathway as well as other biological process (BP). Additionally, they showed enrichment in condensed nuclear chromatin and late endosome membrane among other cellular component (CC). Furthermore, these genes exhibited exodeoxyribonuclease activity, 3’-5’ exonuclease activity, deoxyribonuclease activity, exonuclease activity, and nuclease activity, along with other molecular function (MF) detailed in **Table 1**. We presented the results of GO functional enrichment analysis using bar graphs (**Figure 4A**). Moreover, the enrichment results for BP pathway (**Figure 4B**), CC pathway (**Figure 4C**) and MF pathway (**Figure 4D**) from GO gene functional enrichment analysis were illustrated using a ring network diagram.

**Table 1:** GO and KEGG enrichment analysis results of Co-DEGs.

ONTOLOGY	ID	Description	*P*	geneID	Count
BP	GO:0000076	DNA replication checkpoint signaling	0.003612	RAD9A	1
BP	GO:0031573	mitotic intra-S DNA damage checkpoint signaling	0.003612	RAD9A	1
BP	GO:1902229	regulation of intrinsic apoptotic signaling pathway in response to DNA damage	0.008061	RAD9A	1
BP	GO:2001244	positive regulation of intrinsic apoptotic signaling pathway	0.012917	RAD9A	1
BP	GO:2001235	positive regulation of apoptotic signaling pathway	0.028209	RAD9A	1
CC	GO:0000794	condensed nuclear chromosome	0.015022	RAD9A	1
CC	GO:0031902	late endosome membrane	0.030673	STOML1	1
CC	GO:0000228	nuclear chromosome	0.045742	RAD9A	1
MF	GO:0004529	exodeoxyribonuclease activity	0.005205	RAD9A	1
MF	GO:0008408	3’-5’ exonuclease activity	0.011682	RAD9A	1
MF	GO:0004536	deoxyribonuclease activity	0.012328	RAD9A	1
MF	GO:0004527	exonuclease activity	0.01727	RAD9A	1
MF	GO:0004518	nuclease activity	0.043596	RAD9A	1
KEGG	hsa04218	Cellular senescence	0.019108	RAD9A	1

GO, Gene Ontology; BP, biological process; CC, cellular component; MF, molecular function; Co-DEGs, Common differentially expressed genes.

### 3.4 PPI network and mRNA-RBP, mRNA-miRNA, mRNA-TF, mRNA-drug interaction networks were constructed

**Figure 5:**
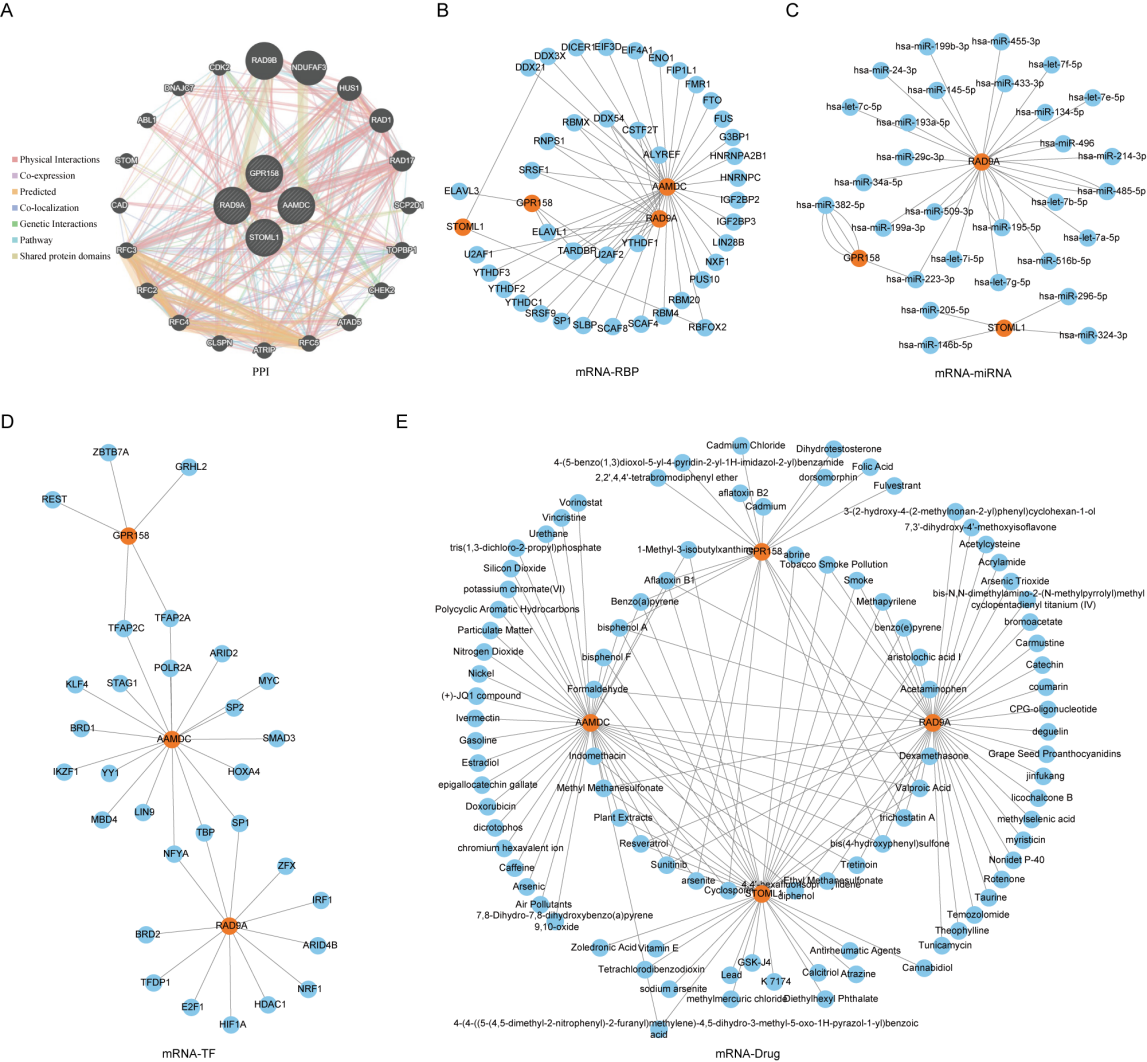
PPI network of Co-DEGs and interaction network of mRNA-RBP, mRNA-miRNA, mRNA-TF and mRNA-drug. **(E)** PPI **(A)**, mRNA-RBP **(B)**, mRNA-miRNA **(C)**, mRNA-TF **(D)**, mRNA-drug **(E)** interaction network of Co-DEGs. In PPI **(A)** network, the inner circle was the Co-DEGs in our study, and the outer circle was the genes with similar functions. The blue circle is miRNA. In the mRNA-RBP **(B)** interaction network, the orange circle is mRNA, and the blue circle is RBP. In the mRNA-miRNA **(C)** interaction network, orange circles are mRNA and blue circles are miRNA. In the mRNA-TF **(D)** interaction network, orange circles are mRNAs and blue circles are transcription factors (TFS). In the mRNA-drug **(E)** interaction network, orange circles are mRNAs and blue circles are drugs or compounds (drugs). RBP, RNA binding protein; TF, Transcription factors; Co-DEGs, Common differentially expressed genes.

We utilized the GeneMANIA website to predicted and constructed an interaction network for 5 Co-DEGs (AAMDC, GPR158, RAD9A, STOML1, STRA13) with similar functions (**Figure 5A**). This allowed us to observe their physical interaction, shared protein domains, gene interaction and other information between them. The details of the interaction relationships are shown in **Table 2**.

**Table 2:** PPI interaction network nodes.

Gene 1	Gene 2	Weight	Network group	Network
CHEK2	RAD9A	0.014346154	Co-expression	Wang-Maris-2006
CHEK2	NDUFAF3	0.013143329	Co-expression	Wang-Maris-2006
RFC4	RAD9A	0.008023581	Co-expression	Wang-Maris-2006
RFC4	TOPBP1	0.004983198	Co-expression	Wang-Maris-2006
RFC4	RFC5	0.010018863	Co-expression	Wang-Maris-2006
RFC3	TOPBP1	0.00891742	Co-expression	Wang-Maris-2006
RFC3	RFC4	0.008753207	Co-expression	Wang-Maris-2006
CDK2	TOPBP1	0.006960052	Co-expression	Wang-Maris-2006
RFC5	ATAD5	0.007891633	Co-expression	Mallon-McKay-2013
RFC4	RFC5	0.005373091	Co-expression	Mallon-McKay-2013
RFC2	CLSPN	0.0158582	Co-expression	Mallon-McKay-2013
RFC2	RFC4	0.00715079	Co-expression	Mallon-McKay-2013
RFC5	TOPBP1	0.009784741	Co-expression	Roth-Zlotnik-2006
RFC4	TOPBP1	0.006803632	Co-expression	Roth-Zlotnik-2006
RFC4	RFC5	0.010834974	Co-expression	Roth-Zlotnik-2006
CDK2	CHEK2	0.020112298	Co-expression	Roth-Zlotnik-2006
CAD	TOPBP1	0.015533725	Co-expression	Ramaswamy-Golub-2001
CHEK2	AAMDC	0.021477731	Co-expression	Innocenti-Brown-2011
CDK2	TOPBP1	0.019024322	Co-expression	Innocenti-Brown-2011
RFC3	RFC4	0.005684063	Co-expression	Alizadeh-Staudt-2000
CDK2	RFC4	0.00596812	Co-expression	Alizadeh-Staudt-2000
CDK2	RFC3	0.006223241	Co-expression	Alizadeh-Staudt-2000
RFC4	TOPBP1	0.007248496	Co-expression	Dobbin-Giordano-2005
RFC2	RFC4	0.010942622	Co-expression	Rieger-Chu-2004
RFC3	TOPBP1	0.008672513	Co-expression	Rieger-Chu-2004
CDK2	CAD	0.013887334	Co-expression	Rieger-Chu-2004
RFC5	TOPBP1	0.016329626	Co-expression	Bild-Nevins-2006 B
RFC4	TOPBP1	0.016536836	Co-expression	Bild-Nevins-2006 B
RFC3	TOPBP1	0.014198861	Co-expression	Bild-Nevins-2006 B
RFC3	RFC4	0.015374002	Co-expression	Bild-Nevins-2006 B
RFC3	RFC2	0.018126553	Co-expression	Bild-Nevins-2006 B
NDUFAF3	STOML1	0.027423456	Co-expression	Burington-Shaughnessy-2008
TOPBP1	RAD1	0.008181981	Co-expression	Burington-Shaughnessy-2008
RFC4	TOPBP1	0.009135751	Co-expression	Burington-Shaughnessy-2008
RFC2	RAD1	0.016552504	Co-expression	Burington-Shaughnessy-2008
RFC2	TOPBP1	0.012052109	Co-expression	Burington-Shaughnessy-2008
RFC2	RFC4	0.01475959	Co-expression	Burington-Shaughnessy-2008
RFC3	TOPBP1	0.008247213	Co-expression	Burington-Shaughnessy-2008
RFC3	RFC5	0.014935831	Co-expression	Burington-Shaughnessy-2008
STOM	STOML1	0.01497809	Co-expression	Burington-Shaughnessy-2008
CDK2	RFC2	0.024156341	Co-expression	Burington-Shaughnessy-2008
CDK2	RFC3	0.014332209	Co-expression	Burington-Shaughnessy-2008
CDK2	RFC3	0.015125908	Co-expression	Boldrick-Relman-2002
TOPBP1	RAD1	0.013603624	Co-expression	Arijs-Rutgeerts-2009
RFC4	RAD1	0.009552398	Co-expression	Arijs-Rutgeerts-2009
RFC4	TOPBP1	0.005602225	Co-expression	Arijs-Rutgeerts-2009
RFC4	CHEK2	0.010617767	Co-expression	Arijs-Rutgeerts-2009
RFC2	RFC4	0.009791195	Co-expression	Arijs-Rutgeerts-2009
RFC3	RFC4	0.012811804	Co-expression	Arijs-Rutgeerts-2009
ATAD5	TOPBP1	0.005691669	Co-expression	Jiang-de Kok-2017
CLSPN	TOPBP1	0.006192454	Co-expression	Jiang-de Kok-2017
CLSPN	ATAD5	0.008432685	Co-expression	Jiang-de Kok-2017
CLSPN	RFC5	0.004001729	Co-expression	Jiang-de Kok-2017
RFC2	RFC5	0.002457261	Co-expression	Jiang-de Kok-2017
RFC2	CLSPN	0.005236648	Co-expression	Jiang-de Kok-2017
RFC3	RFC5	0.008593285	Co-expression	Jiang-de Kok-2017
RFC3	RFC4	0.010327626	Co-expression	Jiang-de Kok-2017
CDK2	TOPBP1	0.004109392	Co-expression	Jiang-de Kok-2017
CDK2	ATAD5	0.005663436	Co-expression	Jiang-de Kok-2017
CDK2	RFC5	0.00261616	Co-expression	Jiang-de Kok-2017
CDK2	CLSPN	0.00629953	Co-expression	Jiang-de Kok-2017
RFC4	TOPBP1	0.004710932	Co-expression	Perou-Botstein-2000
RFC3	RFC4	0.005551286	Co-expression	Perou-Botstein-2000
RFC4	RAD1	0.004494031	Co-expression	Chen-Brown-2002
RFC4	TOPBP1	0.002957351	Co-expression	Chen-Brown-2002
RFC4	TOPBP1	0.005088573	Co-expression	Wang-Cheung-2015
RFC4	RFC5	0.007171569	Co-expression	Wang-Cheung-2015
RFC2	ATAD5	0.012693718	Co-expression	Wang-Cheung-2015
RFC2	RFC5	0.017208459	Co-expression	Wang-Cheung-2015
RFC3	RFC5	0.017267397	Co-expression	Wang-Cheung-2015
RFC3	RFC4	0.006347434	Co-expression	Wang-Cheung-2015
RFC3	RFC2	0.010887823	Co-expression	Wang-Cheung-2015
CDK2	RFC4	0.007397846	Co-expression	Wang-Cheung-2015
CDK2	RFC2	0.011977476	Co-expression	Wang-Cheung-2015
CDK2	RFC3	0.018285148	Co-expression	Wang-Cheung-2015
RFC4	TOPBP1	0.004967705	Co-expression	Ross-Perou-2001
RFC3	RFC4	0.005414083	Co-expression	Ross-Perou-2001
RFC3	TOPBP1	0.017342417	Co-expression	Perou-Botstein-1999
ATRIP	RAD17	0.014442301	Co-localization	Johnson-Shoemaker-2003
CLSPN	ATAD5	0.009249673	Co-localization	Johnson-Shoemaker-2003
RFC4	TOPBP1	0.002514787	Co-localization	Johnson-Shoemaker-2003
RFC4	CHEK2	0.005395827	Co-localization	Johnson-Shoemaker-2003
RFC4	ATAD5	0.001903795	Co-localization	Johnson-Shoemaker-2003
RFC4	ATRIP	0.003973384	Co-localization	Johnson-Shoemaker-2003
RFC4	CLSPN	0.004626494	Co-localization	Johnson-Shoemaker-2003
RFC2	ATAD5	0.007908739	Co-localization	Johnson-Shoemaker-2003
RFC2	RFC4	0.003806144	Co-localization	Johnson-Shoemaker-2003
RFC3	RAD1	0.006628603	Co-localization	Johnson-Shoemaker-2003
RFC3	RAD17	0.003489296	Co-localization	Johnson-Shoemaker-2003
RFC3	TOPBP1	0.003055819	Co-localization	Johnson-Shoemaker-2003
RFC3	CHEK2	0.007366215	Co-localization	Johnson-Shoemaker-2003
RFC3	ATAD5	0.002203455	Co-localization	Johnson-Shoemaker-2003
RFC3	CLSPN	0.005481064	Co-localization	Johnson-Shoemaker-2003
RFC3	RFC4	0.001276434	Co-localization	Johnson-Shoemaker-2003
CAD	ATAD5	0.01136809	Co-localization	Johnson-Shoemaker-2003
CAD	RFC4	0.00532756	Co-localization	Johnson-Shoemaker-2003
CAD	RFC3	0.006873222	Co-localization	Johnson-Shoemaker-2003
CDK2	HUS1	0.006412496	Co-localization	Johnson-Shoemaker-2003
CHEK2	RAD17	0.10440294	Genetic Interactions	Srivas-Ideker-2016
ATAD5	CHEK2	0.056960225	Genetic Interactions	Srivas-Ideker-2016
CDK2	CHEK2	0.71743894	Genetic Interactions	BIOGRID-SMALL-SCALE-STUDIES
RFC5	HUS1	0.14243309	Genetic Interactions	Horlbeck-Gilbert-2018 A
CLSPN	HUS1	0.24364267	Genetic Interactions	Horlbeck-Gilbert-2018 A
RFC4	HUS1	0.13482021	Genetic Interactions	Horlbeck-Gilbert-2018 A
CHEK2	STOML1	0.00483976	Genetic Interactions	Lin-Smith-2010
ATAD5	SCP2D1	0.000972157	Genetic Interactions	Lin-Smith-2010
RFC2	TOPBP1	0.002534529	Genetic Interactions	Lin-Smith-2010
RFC3	AAMDC	0.000455377	Genetic Interactions	Lin-Smith-2010
RFC3	RAD17	0.002090167	Genetic Interactions	Lin-Smith-2010
RFC3	SCP2D1	0.000472877	Genetic Interactions	Lin-Smith-2010
RFC3	ATAD5	0.00042732	Genetic Interactions	Lin-Smith-2010
CAD	RAD9B	0.005111796	Genetic Interactions	Lin-Smith-2010
ABL1	ATAD5	0.000611092	Genetic Interactions	Lin-Smith-2010
HUS1	RAD9A	0.07480734	Pathway	Wu-Stein-2010
HUS1	RAD9B	0.10691969	Pathway	Wu-Stein-2010
RAD1	RAD9A	0.07480734	Pathway	Wu-Stein-2010
RAD1	RAD9B	0.10691969	Pathway	Wu-Stein-2010
RAD1	HUS1	0.070113026	Pathway	Wu-Stein-2010
RAD17	RAD9A	0.090710305	Pathway	Wu-Stein-2010
RAD17	RAD9B	0.12964928	Pathway	Wu-Stein-2010
RAD17	HUS1	0.085018046	Pathway	Wu-Stein-2010
RAD17	RAD1	0.085018046	Pathway	Wu-Stein-2010
RFC5	RAD9A	0.06087736	Pathway	Wu-Stein-2010
RFC5	RAD9B	0.087010026	Pathway	Wu-Stein-2010
RFC5	HUS1	0.05705718	Pathway	Wu-Stein-2010
RFC5	RAD1	0.05705718	Pathway	Wu-Stein-2010
RFC5	RAD17	0.06918672	Pathway	Wu-Stein-2010
ATRIP	RAD9A	0.04536284	Pathway	Wu-Stein-2010
ATRIP	HUS1	0.04251623	Pathway	Wu-Stein-2010
ATRIP	RAD1	0.04251623	Pathway	Wu-Stein-2010
ATRIP	RAD17	0.051554576	Pathway	Wu-Stein-2010
CLSPN	ATRIP	0.040571287	Pathway	Wu-Stein-2010
RFC4	RAD9A	0.06087736	Pathway	Wu-Stein-2010
RFC4	RAD9B	0.087010026	Pathway	Wu-Stein-2010
RFC4	HUS1	0.05705718	Pathway	Wu-Stein-2010
RFC4	RAD1	0.05705718	Pathway	Wu-Stein-2010
RFC4	RAD17	0.06918672	Pathway	Wu-Stein-2010
RFC4	RFC5	0.046432484	Pathway	Wu-Stein-2010
RFC2	RAD9A	0.06087736	Pathway	Wu-Stein-2010
RFC2	RAD9B	0.087010026	Pathway	Wu-Stein-2010
RFC2	HUS1	0.05705718	Pathway	Wu-Stein-2010
RFC2	RAD1	0.05705718	Pathway	Wu-Stein-2010
RFC2	RAD17	0.06918672	Pathway	Wu-Stein-2010
RFC2	RFC5	0.046432484	Pathway	Wu-Stein-2010
RFC2	RFC4	0.046432484	Pathway	Wu-Stein-2010
RFC3	RAD9A	0.06087736	Pathway	Wu-Stein-2010
RFC3	RAD9B	0.087010026	Pathway	Wu-Stein-2010
RFC3	HUS1	0.05705718	Pathway	Wu-Stein-2010
RFC3	RAD1	0.05705718	Pathway	Wu-Stein-2010
RFC3	RAD17	0.06918672	Pathway	Wu-Stein-2010
RFC3	RFC5	0.046432484	Pathway	Wu-Stein-2010
RFC3	RFC4	0.046432484	Pathway	Wu-Stein-2010
RFC3	RFC2	0.046432484	Pathway	Wu-Stein-2010
CDK2	ATRIP	0.011560454	Pathway	Wu-Stein-2010
CDK2	CLSPN	0.018192103	Pathway	Wu-Stein-2010
CDK2	ABL1	0.010743902	Pathway	Wu-Stein-2010
HUS1	RAD9A	0.021693664	Pathway	NCI_NATURE
RAD1	RAD9A	0.02129535	Pathway	NCI_NATURE
RAD1	HUS1	0.021693664	Pathway	NCI_NATURE
RAD17	RAD9A	0.023137692	Pathway	NCI_NATURE
RAD17	HUS1	0.023570465	Pathway	NCI_NATURE
RAD17	RAD1	0.023137692	Pathway	NCI_NATURE
TOPBP1	RAD9A	0.022310786	Pathway	NCI_NATURE
TOPBP1	HUS1	0.022728093	Pathway	NCI_NATURE
TOPBP1	RAD1	0.022310786	Pathway	NCI_NATURE
TOPBP1	RAD17	0.02424098	Pathway	NCI_NATURE
RFC5	RAD9A	0.024917956	Pathway	NCI_NATURE
RFC5	HUS1	0.02538403	Pathway	NCI_NATURE
RFC5	RAD1	0.024917956	Pathway	NCI_NATURE
RFC5	RAD17	0.027073704	Pathway	NCI_NATURE
RFC5	TOPBP1	0.026106132	Pathway	NCI_NATURE
ATRIP	RAD9A	0.021977346	Pathway	NCI_NATURE
ATRIP	HUS1	0.022388417	Pathway	NCI_NATURE
ATRIP	RAD1	0.021977346	Pathway	NCI_NATURE
ATRIP	RAD17	0.023878692	Pathway	NCI_NATURE
ATRIP	TOPBP1	0.023025304	Pathway	NCI_NATURE
ATRIP	RFC5	0.02571597	Pathway	NCI_NATURE
CLSPN	RAD9A	0.03931835	Pathway	NCI_NATURE
CLSPN	HUS1	0.040053774	Pathway	NCI_NATURE
CLSPN	RAD1	0.03931835	Pathway	NCI_NATURE
CLSPN	RAD17	0.042719934	Pathway	NCI_NATURE
CLSPN	TOPBP1	0.04119319	Pathway	NCI_NATURE
CLSPN	RFC5	0.0460069	Pathway	NCI_NATURE
CLSPN	ATRIP	0.040577546	Pathway	NCI_NATURE
RFC4	RAD9A	0.025497014	Pathway	NCI_NATURE
RFC4	HUS1	0.025973918	Pathway	NCI_NATURE
RFC4	RAD1	0.025497014	Pathway	NCI_NATURE
RFC4	RAD17	0.027702859	Pathway	NCI_NATURE
RFC4	TOPBP1	0.026712801	Pathway	NCI_NATURE
RFC4	RFC5	0.029834377	Pathway	NCI_NATURE
RFC4	ATRIP	0.026313573	Pathway	NCI_NATURE
RFC4	CLSPN	0.04707603	Pathway	NCI_NATURE
RFC2	RAD9A	0.024917956	Pathway	NCI_NATURE
RFC2	HUS1	0.02538403	Pathway	NCI_NATURE
RFC2	RAD1	0.024917956	Pathway	NCI_NATURE
RFC2	RAD17	0.027073704	Pathway	NCI_NATURE
RFC2	TOPBP1	0.026106132	Pathway	NCI_NATURE
RFC2	RFC5	0.029156813	Pathway	NCI_NATURE
RFC2	ATRIP	0.02571597	Pathway	NCI_NATURE
RFC2	CLSPN	0.0460069	Pathway	NCI_NATURE
RFC2	RFC4	0.029834377	Pathway	NCI_NATURE
RFC3	RAD9A	0.024917956	Pathway	NCI_NATURE
RFC3	HUS1	0.02538403	Pathway	NCI_NATURE
RFC3	RAD1	0.024917956	Pathway	NCI_NATURE
RFC3	RAD17	0.027073704	Pathway	NCI_NATURE
RFC3	TOPBP1	0.026106132	Pathway	NCI_NATURE
RFC3	RFC5	0.029156813	Pathway	NCI_NATURE
RFC3	ATRIP	0.02571597	Pathway	NCI_NATURE
RFC3	CLSPN	0.0460069	Pathway	NCI_NATURE
RFC3	RFC4	0.029834377	Pathway	NCI_NATURE
RFC3	RFC2	0.029156813	Pathway	NCI_NATURE
CDK2	ATRIP	0.036545698	Pathway	NCI_NATURE
CDK2	ABL1	0.049224008	Pathway	NCI_NATURE
CDK2	CLSPN	0.025033776	Pathway	REACTOME
ABL1	RAD9A	0.087588504	Physical Interactions	Colicelli-2010
ABL1	TOPBP1	0.087588504	Physical Interactions	Colicelli-2010
HUS1	RAD9A	0.11682344	Physical Interactions	IREF-reactome
HUS1	RAD9B	0.11682344	Physical Interactions	IREF-reactome
RAD1	RAD9A	0.11682344	Physical Interactions	IREF-reactome
RAD1	RAD9B	0.11682344	Physical Interactions	IREF-reactome
RAD1	HUS1	0.10782476	Physical Interactions	IREF-reactome
RAD17	RAD9A	0.11682344	Physical Interactions	IREF-reactome
RAD17	RAD9B	0.11682344	Physical Interactions	IREF-reactome
RAD17	HUS1	0.10782476	Physical Interactions	IREF-reactome
RAD17	RAD1	0.10782476	Physical Interactions	IREF-reactome
RFC5	RAD9A	0.0553185	Physical Interactions	IREF-reactome
RFC5	RAD9B	0.0553185	Physical Interactions	IREF-reactome
RFC5	HUS1	0.05105742	Physical Interactions	IREF-reactome
RFC5	RAD1	0.05105742	Physical Interactions	IREF-reactome
RFC5	RAD17	0.05105742	Physical Interactions	IREF-reactome
ATRIP	RAD9A	0.061430365	Physical Interactions	IREF-reactome
ATRIP	RAD9B	0.061430365	Physical Interactions	IREF-reactome
ATRIP	HUS1	0.056698505	Physical Interactions	IREF-reactome
ATRIP	RAD1	0.056698505	Physical Interactions	IREF-reactome
ATRIP	RAD17	0.056698505	Physical Interactions	IREF-reactome
ATRIP	RFC5	0.026848003	Physical Interactions	IREF-reactome
CLSPN	ATRIP	0.036601167	Physical Interactions	IREF-reactome
RFC4	RAD9A	0.0553185	Physical Interactions	IREF-reactome
RFC4	RAD9B	0.0553185	Physical Interactions	IREF-reactome
RFC4	HUS1	0.05105742	Physical Interactions	IREF-reactome
RFC4	RAD1	0.05105742	Physical Interactions	IREF-reactome
RFC4	RAD17	0.05105742	Physical Interactions	IREF-reactome
RFC4	RFC5	0.024176827	Physical Interactions	IREF-reactome
RFC4	ATRIP	0.026848003	Physical Interactions	IREF-reactome
RFC2	RAD9A	0.0553185	Physical Interactions	IREF-reactome
RFC2	RAD9B	0.0553185	Physical Interactions	IREF-reactome
RFC2	HUS1	0.05105742	Physical Interactions	IREF-reactome
RFC2	RAD1	0.05105742	Physical Interactions	IREF-reactome
RFC2	RAD17	0.05105742	Physical Interactions	IREF-reactome
RFC2	RFC5	0.024176827	Physical Interactions	IREF-reactome
RFC2	ATRIP	0.026848003	Physical Interactions	IREF-reactome
RFC2	RFC4	0.024176827	Physical Interactions	IREF-reactome
RFC3	RAD9A	0.0553185	Physical Interactions	IREF-reactome
RFC3	RAD9B	0.0553185	Physical Interactions	IREF-reactome
RFC3	HUS1	0.05105742	Physical Interactions	IREF-reactome
RFC3	RAD1	0.05105742	Physical Interactions	IREF-reactome
RFC3	RAD17	0.05105742	Physical Interactions	IREF-reactome
RFC3	RFC5	0.024176827	Physical Interactions	IREF-reactome
RFC3	ATRIP	0.026848003	Physical Interactions	IREF-reactome
RFC3	RFC4	0.024176827	Physical Interactions	IREF-reactome
RFC3	RFC2	0.024176827	Physical Interactions	IREF-reactome
CDK2	ATRIP	0.012541235	Physical Interactions	IREF-reactome
CDK2	CLSPN	0.015396093	Physical Interactions	IREF-reactome
HUS1	RAD9A	0.11682344	Physical Interactions	Vastrik-Stein-2007
HUS1	RAD9B	0.11682344	Physical Interactions	Vastrik-Stein-2007
RAD1	RAD9A	0.11682344	Physical Interactions	Vastrik-Stein-2007
RAD1	RAD9B	0.11682344	Physical Interactions	Vastrik-Stein-2007
RAD1	HUS1	0.10782476	Physical Interactions	Vastrik-Stein-2007
RAD17	RAD9A	0.11682344	Physical Interactions	Vastrik-Stein-2007
RAD17	RAD9B	0.11682344	Physical Interactions	Vastrik-Stein-2007
RAD17	HUS1	0.10782476	Physical Interactions	Vastrik-Stein-2007
RAD17	RAD1	0.10782476	Physical Interactions	Vastrik-Stein-2007
RFC5	RAD9A	0.0553185	Physical Interactions	Vastrik-Stein-2007
RFC5	RAD9B	0.0553185	Physical Interactions	Vastrik-Stein-2007
RFC5	HUS1	0.05105742	Physical Interactions	Vastrik-Stein-2007
RFC5	RAD1	0.05105742	Physical Interactions	Vastrik-Stein-2007
RFC5	RAD17	0.05105742	Physical Interactions	Vastrik-Stein-2007
ATRIP	RAD9A	0.061430365	Physical Interactions	Vastrik-Stein-2007
ATRIP	RAD9B	0.061430365	Physical Interactions	Vastrik-Stein-2007
ATRIP	HUS1	0.056698505	Physical Interactions	Vastrik-Stein-2007
ATRIP	RAD1	0.056698505	Physical Interactions	Vastrik-Stein-2007
ATRIP	RAD17	0.056698505	Physical Interactions	Vastrik-Stein-2007
ATRIP	RFC5	0.026848003	Physical Interactions	Vastrik-Stein-2007
CLSPN	ATRIP	0.036601167	Physical Interactions	Vastrik-Stein-2007
RFC4	RAD9A	0.0553185	Physical Interactions	Vastrik-Stein-2007
RFC4	RAD9B	0.0553185	Physical Interactions	Vastrik-Stein-2007
RFC4	HUS1	0.05105742	Physical Interactions	Vastrik-Stein-2007
RFC4	RAD1	0.05105742	Physical Interactions	Vastrik-Stein-2007
RFC4	RAD17	0.05105742	Physical Interactions	Vastrik-Stein-2007
RFC4	RFC5	0.024176827	Physical Interactions	Vastrik-Stein-2007
RFC4	ATRIP	0.026848003	Physical Interactions	Vastrik-Stein-2007
RFC2	RAD9A	0.0553185	Physical Interactions	Vastrik-Stein-2007
RFC2	RAD9B	0.0553185	Physical Interactions	Vastrik-Stein-2007
RFC2	HUS1	0.05105742	Physical Interactions	Vastrik-Stein-2007
RFC2	RAD1	0.05105742	Physical Interactions	Vastrik-Stein-2007
RFC2	RAD17	0.05105742	Physical Interactions	Vastrik-Stein-2007
RFC2	RFC5	0.024176827	Physical Interactions	Vastrik-Stein-2007
RFC2	ATRIP	0.026848003	Physical Interactions	Vastrik-Stein-2007
RFC2	RFC4	0.024176827	Physical Interactions	Vastrik-Stein-2007
RFC3	RAD9A	0.0553185	Physical Interactions	Vastrik-Stein-2007
RFC3	RAD9B	0.0553185	Physical Interactions	Vastrik-Stein-2007
RFC3	HUS1	0.05105742	Physical Interactions	Vastrik-Stein-2007
RFC3	RAD1	0.05105742	Physical Interactions	Vastrik-Stein-2007
RFC3	RAD17	0.05105742	Physical Interactions	Vastrik-Stein-2007
RFC3	RFC5	0.024176827	Physical Interactions	Vastrik-Stein-2007
RFC3	ATRIP	0.026848003	Physical Interactions	Vastrik-Stein-2007
RFC3	RFC4	0.024176827	Physical Interactions	Vastrik-Stein-2007
RFC3	RFC2	0.024176827	Physical Interactions	Vastrik-Stein-2007
CDK2	ATRIP	0.012541235	Physical Interactions	Vastrik-Stein-2007
CDK2	CLSPN	0.015396093	Physical Interactions	Vastrik-Stein-2007
DNAJC7	CAD	0.04957664	Physical Interactions	Hein-Mann-2015
RAD9B	RAD9A	0.40262133	Physical Interactions	IREF-quickgo
HUS1	RAD9A	0.11029065	Physical Interactions	IREF-quickgo
RAD1	RAD9A	0.40262133	Physical Interactions	IREF-quickgo
RAD17	RAD9A	0.111976705	Physical Interactions	IREF-quickgo
RAD17	HUS1	0.26391461	Physical Interactions	IREF-quickgo
RFC4	ATAD5	0.40693566	Physical Interactions	IREF-quickgo
RFC4	RFC5	0.60778123	Physical Interactions	IREF-quickgo
RFC3	RFC4	0.60778123	Physical Interactions	IREF-quickgo
CAD	RAD9A	0.40262133	Physical Interactions	IREF-quickgo
ABL1	RAD9A	0.052999433	Physical Interactions	IREF-quickgo
TOPBP1	RAD9A	0.34152973	Physical Interactions	IREF-dip
CHEK2	RAD9A	0.44381043	Physical Interactions	IREF-dip
RFC5	RAD17	0.5040152	Physical Interactions	IREF-dip
RFC4	RAD17	0.34456268	Physical Interactions	IREF-dip
RFC2	RAD17	0.5040152	Physical Interactions	IREF-dip
RFC3	RAD17	0.5040152	Physical Interactions	IREF-dip
RFC4	RFC5	0.18730119	Physical Interactions	Wan-Emili-2015
RFC2	RFC5	0.16978356	Physical Interactions	Wan-Emili-2015
RFC2	RFC4	0.18617004	Physical Interactions	Wan-Emili-2015
RFC3	RFC5	0.21160097	Physical Interactions	Wan-Emili-2015
RFC3	RFC4	0.23202343	Physical Interactions	Wan-Emili-2015
RFC3	RFC2	0.21032307	Physical Interactions	Wan-Emili-2015
RFC4	RFC5	0.16233994	Physical Interactions	Havugimana-Emili-2012
RFC2	RFC5	0.16630344	Physical Interactions	Havugimana-Emili-2012
RFC2	RFC4	0.15051839	Physical Interactions	Havugimana-Emili-2012
RFC3	RFC5	0.15458822	Physical Interactions	Havugimana-Emili-2012
RFC3	RFC4	0.13991514	Physical Interactions	Havugimana-Emili-2012
RFC3	RFC2	0.14333117	Physical Interactions	Havugimana-Emili-2012
RFC2	RFC4	0.62289274	Physical Interactions	IREF-uniprotpp
CDK2	STOML1	0.5439336	Physical Interactions	IREF-uniprotpp
HUS1	RAD9A	0.025538957	Physical Interactions	BIOGRID-SMALL-SCALE-STUDIES
HUS1	RAD9B	0.12031516	Physical Interactions	BIOGRID-SMALL-SCALE-STUDIES
RAD1	RAD9A	0.02505026	Physical Interactions	BIOGRID-SMALL-SCALE-STUDIES
RAD1	RAD9B	0.11801288	Physical Interactions	BIOGRID-SMALL-SCALE-STUDIES
RAD1	HUS1	0.032714687	Physical Interactions	BIOGRID-SMALL-SCALE-STUDIES
RAD17	RAD9A	0.027845085	Physical Interactions	BIOGRID-SMALL-SCALE-STUDIES
RAD17	RAD9B	0.13117944	Physical Interactions	BIOGRID-SMALL-SCALE-STUDIES
RAD17	HUS1	0.036364622	Physical Interactions	BIOGRID-SMALL-SCALE-STUDIES
RAD17	RAD1	0.03566877	Physical Interactions	BIOGRID-SMALL-SCALE-STUDIES
TOPBP1	RAD9A	0.015908115	Physical Interactions	BIOGRID-SMALL-SCALE-STUDIES
TOPBP1	HUS1	0.020775393	Physical Interactions	BIOGRID-SMALL-SCALE-STUDIES
TOPBP1	RAD1	0.020377846	Physical Interactions	BIOGRID-SMALL-SCALE-STUDIES
RFC5	RAD9A	0.040328022	Physical Interactions	BIOGRID-SMALL-SCALE-STUDIES
RFC5	HUS1	0.05266687	Physical Interactions	BIOGRID-SMALL-SCALE-STUDIES
RFC5	RAD1	0.051659066	Physical Interactions	BIOGRID-SMALL-SCALE-STUDIES
RFC5	RAD17	0.057422604	Physical Interactions	BIOGRID-SMALL-SCALE-STUDIES
RFC5	ATAD5	0.120061316	Physical Interactions	BIOGRID-SMALL-SCALE-STUDIES
ATRIP	TOPBP1	0.021319931	Physical Interactions	BIOGRID-SMALL-SCALE-STUDIES
CLSPN	RAD9A	0.029172774	Physical Interactions	BIOGRID-SMALL-SCALE-STUDIES
CLSPN	RAD17	0.041538775	Physical Interactions	BIOGRID-SMALL-SCALE-STUDIES
RFC4	RAD17	0.043366734	Physical Interactions	BIOGRID-SMALL-SCALE-STUDIES
RFC4	ATAD5	0.090672776	Physical Interactions	BIOGRID-SMALL-SCALE-STUDIES
RFC4	RFC5	0.06280802	Physical Interactions	BIOGRID-SMALL-SCALE-STUDIES
RFC2	RAD17	0.055125087	Physical Interactions	BIOGRID-SMALL-SCALE-STUDIES
RFC2	RFC5	0.07983764	Physical Interactions	BIOGRID-SMALL-SCALE-STUDIES
RFC2	RFC4	0.060295027	Physical Interactions	BIOGRID-SMALL-SCALE-STUDIES
RFC3	RAD17	0.057611387	Physical Interactions	BIOGRID-SMALL-SCALE-STUDIES
RFC3	ATAD5	0.12045604	Physical Interactions	BIOGRID-SMALL-SCALE-STUDIES
RFC3	RFC4	0.063014515	Physical Interactions	BIOGRID-SMALL-SCALE-STUDIES
CAD	RAD9A	0.044138934	Physical Interactions	BIOGRID-SMALL-SCALE-STUDIES
ABL1	RAD9A	0.007967228	Physical Interactions	BIOGRID-SMALL-SCALE-STUDIES
ABL1	TOPBP1	0.006481168	Physical Interactions	BIOGRID-SMALL-SCALE-STUDIES
DNAJC7	RAD9A	0.047350135	Physical Interactions	BIOGRID-SMALL-SCALE-STUDIES
DNAJC7	HUS1	0.06183748	Physical Interactions	BIOGRID-SMALL-SCALE-STUDIES
DNAJC7	RAD1	0.060654193	Physical Interactions	BIOGRID-SMALL-SCALE-STUDIES
CDK2	STOML1	0.11462871	Physical Interactions	BIOGRID-SMALL-SCALE-STUDIES
HUS1	RAD9A	0.20159602	Physical Interactions	Huttlin-Harper-2017
RAD1	RAD9A	0.33778113	Physical Interactions	Huttlin-Harper-2017
RAD1	HUS1	0.13003722	Physical Interactions	Huttlin-Harper-2017
RAD17	HUS1	0.14008722	Physical Interactions	Huttlin-Harper-2017
RFC5	RAD17	0.13364537	Physical Interactions	Huttlin-Harper-2017
RFC5	ATAD5	0.30299598	Physical Interactions	Huttlin-Harper-2017
RFC4	RAD1	0.08883654	Physical Interactions	Huttlin-Harper-2017
RFC4	RAD17	0.09570232	Physical Interactions	Huttlin-Harper-2017
RFC4	ATAD5	0.21697284	Physical Interactions	Huttlin-Harper-2017
RFC4	RFC5	0.05058172	Physical Interactions	Huttlin-Harper-2017
RFC2	RAD17	0.3181012	Physical Interactions	Huttlin-Harper-2017
RFC2	RFC5	0.16812661	Physical Interactions	Huttlin-Harper-2017
RFC2	RFC4	0.120394036	Physical Interactions	Huttlin-Harper-2017
RFC3	RAD17	0.28210336	Physical Interactions	Huttlin-Harper-2017
RFC3	RFC5	0.1491006	Physical Interactions	Huttlin-Harper-2017
RFC3	RFC4	0.10676968	Physical Interactions	Huttlin-Harper-2017
RAD17	RAD9A	0.22399561	Physical Interactions	IREF-mint
TOPBP1	RAD9A	0.16913728	Physical Interactions	IREF-mint
ATRIP	TOPBP1	0.19219229	Physical Interactions	IREF-mint
CLSPN	RAD9A	0.3434043	Physical Interactions	IREF-mint
HUS1	RAD9A	0.35929573	Physical Interactions	IREF-bind-translation
DNAJC7	RAD9A	0.21179481	Physical Interactions	IREF-bind-translation
DNAJC7	HUS1	0.2709802	Physical Interactions	IREF-bind-translation
DNAJC7	RAD1	0.3865471	Physical Interactions	IREF-bind-translation
DNAJC7	RAD9A	0.6614378	Physical Interactions	IREF-bind
DNAJC7	HUS1	0.6614378	Physical Interactions	IREF-bind
HUS1	RAD9A	0.2703634	Physical Interactions	Huttlin-Gygi-2015
RAD1	RAD9A	0.49807796	Physical Interactions	Huttlin-Gygi-2015
RAD1	HUS1	0.17728211	Physical Interactions	Huttlin-Gygi-2015
RAD17	HUS1	0.5217113	Physical Interactions	Huttlin-Gygi-2015
RAD17	RAD9A	0.20048356	Physical Interactions	Rual-Vidal-2005
RAD17	HUS1	0.35603815	Physical Interactions	Rual-Vidal-2005
RAD17	RAD1	0.2973175	Physical Interactions	Rual-Vidal-2005
RFC4	RAD17	0.2697884	Physical Interactions	Rual-Vidal-2005
DNAJC7	RAD9A	0.35261863	Physical Interactions	Rual-Vidal-2005
DNAJC7	RAD1	0.52293414	Physical Interactions	Rual-Vidal-2005
STOM	STOML1	0.46869963	Physical Interactions	IREF-matrixdb
RAD17	RAD9A	0.20741317	Physical Interactions	IREF-spike
RAD17	HUS1	0.3965527	Physical Interactions	IREF-spike
RAD17	RAD1	0.39012912	Physical Interactions	IREF-spike
TOPBP1	RAD9A	0.16398469	Physical Interactions	IREF-spike
RFC4	RAD17	0.28198662	Physical Interactions	IREF-spike
ABL1	RAD9A	0.011101241	Physical Interactions	IREF-spike
ABL1	TOPBP1	0.007515909	Physical Interactions	IREF-spike
DNAJC7	RAD9A	0.1892795	Physical Interactions	IREF-spike
DNAJC7	RAD1	0.35602096	Physical Interactions	IREF-spike
RFC4	RFC5	0.5	Physical Interactions	Chen-Yu-2018
HUS1	RAD9A	0.6371664	Physical Interactions	IREF-huri
RFC4	RFC5	0.15941317	Physical Interactions	IREF-huri
RFC3	RFC4	0.3826219	Physical Interactions	IREF-huri
RAD1	HUS1	0.058314063	Physical Interactions	IREF-biogrid
TOPBP1	HUS1	0.028094139	Physical Interactions	IREF-biogrid
TOPBP1	RAD1	0.039620515	Physical Interactions	IREF-biogrid
ATRIP	TOPBP1	0.019734733	Physical Interactions	IREF-biogrid
RFC4	RAD1	0.03167883	Physical Interactions	IREF-biogrid
RFC4	ATAD5	0.04825461	Physical Interactions	IREF-biogrid
ABL1	TOPBP1	0.010033981	Physical Interactions	IREF-biogrid
RFC4	RFC5	0.056360006	Predicted	I2D-BioGRID-Yeast2Human
RFC2	RFC5	0.040982828	Predicted	I2D-BioGRID-Yeast2Human
RFC2	RFC4	0.05895046	Predicted	I2D-BioGRID-Yeast2Human
RFC3	CHEK2	0.013392916	Predicted	I2D-BioGRID-Yeast2Human
RFC3	RFC5	0.028315607	Predicted	I2D-BioGRID-Yeast2Human
RFC3	RFC4	0.040729694	Predicted	I2D-BioGRID-Yeast2Human
RFC3	RFC2	0.029617067	Predicted	I2D-BioGRID-Yeast2Human
CDK2	CHEK2	0.006513496	Predicted	I2D-BioGRID-Yeast2Human
CDK2	RFC3	0.009951877	Predicted	I2D-BioGRID-Yeast2Human
RFC4	RFC5	0.2783674	Predicted	I2D-vonMering-Bork-2002-High-Yeast2Human
RFC2	RFC5	0.2783674	Predicted	I2D-vonMering-Bork-2002-High-Yeast2Human
RFC2	RFC4	0.21170229	Predicted	I2D-vonMering-Bork-2002-High-Yeast2Human
RFC3	RFC5	0.36602542	Predicted	I2D-vonMering-Bork-2002-High-Yeast2Human
RFC3	RFC4	0.2783674	Predicted	I2D-vonMering-Bork-2002-High-Yeast2Human
RFC3	RFC2	0.2783674	Predicted	I2D-vonMering-Bork-2002-High-Yeast2Human
RFC3	RFC5	0.70710677	Predicted	I2D-Li-Vidal-2004-interolog-Worm2Human
RFC3	RFC2	0.70710677	Predicted	I2D-Li-Vidal-2004-interolog-Worm2Human
RFC4	RFC5	1	Predicted	I2D-Tarassov-PCA-Yeast2Human
TOPBP1	RAD9A	0.13623849	Predicted	Wu-Stein-2010
CHEK2	RAD9A	0.058454573	Predicted	Wu-Stein-2010
CLSPN	RAD9A	0.2501647	Predicted	Wu-Stein-2010
ATRIP	TOPBP1	0.8660254	Predicted	I2D-IntAct-Mouse2Human
ATAD5	RAD9A	0.85483515	Predicted	I2D-BioGRID-Mouse2Human
RFC4	RFC5	0.11216343	Predicted	Stuart-Kim-2003
RFC2	RAD17	0.042141847	Predicted	Stuart-Kim-2003
RFC4	RFC5	0.37288517	Predicted	I2D-Yu-Vidal-2008-GoldStd-Yeast2Human
RFC2	RFC5	0.4626682	Predicted	I2D-Yu-Vidal-2008-GoldStd-Yeast2Human
RFC3	RFC4	0.7962252	Predicted	I2D-Yu-Vidal-2008-GoldStd-Yeast2Human
RFC3	RFC5	0.36045358	Predicted	I2D-Krogan-Greenblatt-2006-Core-Yeast2Human
RFC3	RFC4	0.36045358	Predicted	I2D-Krogan-Greenblatt-2006-Core-Yeast2Human
HUS1	RAD9A	0.76536685	Predicted	I2D-BioGRID-Fly2Human
RAD1	HUS1	0.50372267	Predicted	I2D-BioGRID-Fly2Human
RFC4	RFC5	0.145126	Predicted	I2D-BioGRID-Fly2Human
RFC2	RFC5	0.12062031	Predicted	I2D-BioGRID-Fly2Human
RFC2	RFC4	0.12649229	Predicted	I2D-BioGRID-Fly2Human
RFC3	RFC4	0.17079872	Predicted	I2D-BioGRID-Fly2Human
RFC4	RFC5	0.104152426	Predicted	I2D-IntAct-Yeast2Human
RFC2	RFC5	0.08644463	Predicted	I2D-IntAct-Yeast2Human
RFC2	RFC4	0.112059094	Predicted	I2D-IntAct-Yeast2Human
RFC3	RFC5	0.12578438	Predicted	I2D-IntAct-Yeast2Human
RFC3	RFC4	0.16305566	Predicted	I2D-IntAct-Yeast2Human
RFC3	RFC2	0.13533324	Predicted	I2D-IntAct-Yeast2Human
CAD	RFC2	0.014449741	Predicted	I2D-IntAct-Yeast2Human
CDK2	CAD	0.010407695	Predicted	I2D-IntAct-Yeast2Human
RFC4	RFC5	0.36227605	Predicted	I2D-BIND-Yeast2Human
RFC2	RFC5	0.29896703	Predicted	I2D-BIND-Yeast2Human
RFC3	RFC4	0.58578646	Predicted	I2D-BIND-Yeast2Human
RAD9B	RAD9A	1	Shared protein domains	INTERPRO
NDUFAF3	AAMDC	1	Shared protein domains	INTERPRO
SCP2D1	STOML1	0.19736497	Shared protein domains	INTERPRO
RFC5	ATAD5	0.01477202	Shared protein domains	INTERPRO
RFC4	ATAD5	0.014772342	Shared protein domains	INTERPRO
RFC4	RFC5	0.043304753	Shared protein domains	INTERPRO
RFC2	ATAD5	0.014772328	Shared protein domains	INTERPRO
RFC2	RFC5	0.043304916	Shared protein domains	INTERPRO
RFC2	RFC4	0.04330586	Shared protein domains	INTERPRO
RFC3	ATAD5	0.011504605	Shared protein domains	INTERPRO
RFC3	RFC5	0.03275371	Shared protein domains	INTERPRO
RFC3	RFC4	0.03275442	Shared protein domains	INTERPRO
RFC3	RFC2	0.032754466	Shared protein domains	INTERPRO
STOM	STOML1	0.096265905	Shared protein domains	INTERPRO
RAD9B	RAD9A	1	Shared protein domains	PFAM
NDUFAF3	AAMDC	1	Shared protein domains	PFAM
SCP2D1	STOML1	0.20368233	Shared protein domains	PFAM
RFC5	ATAD5	0.022292946	Shared protein domains	PFAM
RFC4	ATAD5	0.022292946	Shared protein domains	PFAM
RFC4	RFC5	0.066983745	Shared protein domains	PFAM
RFC2	ATAD5	0.022292852	Shared protein domains	PFAM
RFC2	RFC5	0.06698409	Shared protein domains	PFAM
RFC2	RFC4	0.06698409	Shared protein domains	PFAM
STOM	STOML1	0.07659206	Shared protein domains	PFAM

We utilized the ENCORI database to predict RNA binding proteins (RBP) that interacted with five Co-DEGs. Following rigorous screening, we identified four Co-DEGs (AAMDC, GPR158, RAD9A, STOML1), resulting in a total of 41 RBP molecules forming 56 pairs of mRNA-RBP interaction relationships (**Figure 5B**). The specific mRNA-RBP interaction relationships are shown in **Table 3**.

**Table 3:** mRNA-RBP interaction network nodes.

mRNA	RBP	mRNA	RBP	mRNA	RBP
AAMDC	ALYREF	AAMDC	LIN28B	AAMDC	YTHDF3
AAMDC	CSTF2T	AAMDC	NXF1	GPR158	ELAVL1
AAMDC	DDX21	AAMDC	PUS10	GPR158	ELAVL3
AAMDC	DDX3X	AAMDC	RBFOX2	GPR158	TARDBP
AAMDC	DDX54	AAMDC	RBM20	GPR158	U2AF2
AAMDC	DICER1	AAMDC	RBM4	RAD9A	ALYREF
AAMDC	EIF3D	AAMDC	RBMX	RAD9A	CSTF2T
AAMDC	EIF4A1	AAMDC	RNPS1	RAD9A	DDX54
AAMDC	ELAVL1	AAMDC	SCAF4	RAD9A	ELAVL1
AAMDC	ENO1	AAMDC	SCAF8	RAD9A	RBMX
AAMDC	FIP1L1	AAMDC	SLBP	RAD9A	RNPS1
AAMDC	FMR1	AAMDC	SP1	RAD9A	SRSF1
AAMDC	FTO	AAMDC	SRSF1	RAD9A	TARDBP
AAMDC	FUS	AAMDC	SRSF9	RAD9A	U2AF1
AAMDC	G3BP1	AAMDC	TARDBP	RAD9A	U2AF2
AAMDC	HNRNPA2B1	AAMDC	U2AF2	RAD9A	YTHDF1
AAMDC	HNRNPC	AAMDC	YTHDC1	STOML1	DDX3X
AAMDC	IGF2BP2	AAMDC	YTHDF1	STOML1	RBFOX2
AAMDC	IGF2BP3	AAMDC	YTHDF2		

RBP, RNA binding protein.

ENCOR database was utilized for the prediction of RNA binding proteins (RBP) that interact with 5 Co-DEGs. Following screening, a total of 3 Co-DEGs (GPR158, RAD9A, STOML1) and 29 miRNA molecules were identified. This resulted in the formation of 39 pairs of mRNA-miRNA interaction relationships (**Figure 5C**), and the specific mRNA-miRNA interaction relationships are shown in **Table 4**.

**Table 4:** mRNA-miRNA interaction network nodes.

miRNA	mRNA	miRNA	mRNA	miRNA	mRNA
hsa-miR-223-3p	GPR158	hsa-miR-214-3p	RAD9A	hsa-miR-485-5p	RAD9A
hsa-miR-382-5p	GPR158	hsa-miR-223-3p	RAD9A	hsa-miR-496	RAD9A
hsa-miR-382-5p	GPR158	hsa-let-7g-5p	RAD9A	hsa-miR-496	RAD9A
hsa-miR-382-5p	GPR158	hsa-let-7i-5p	RAD9A	hsa-miR-516b-5p	RAD9A
hsa-miR-382-5p	GPR158	hsa-miR-145-5p	RAD9A	hsa-miR-509-3p	RAD9A
hsa-let-7a-5p	RAD9A	hsa-miR-134-5p	RAD9A	hsa-miR-509-3p	RAD9A
hsa-let-7b-5p	RAD9A	hsa-miR-195-5p	RAD9A	hsa-miR-199b-3p	RAD9A
hsa-let-7c-5p	RAD9A	hsa-miR-195-5p	RAD9A	hsa-miR-193a-5p	RAD9A
hsa-let-7e-5p	RAD9A	hsa-miR-195-5p	RAD9A	hsa-miR-455-3p	RAD9A
hsa-let-7f-5p	RAD9A	hsa-miR-29c-3p	RAD9A	hsa-miR-205-5p	STOML1
hsa-miR-24-3p	RAD9A	hsa-miR-433-3p	RAD9A	hsa-miR-296-5p	STOML1
hsa-miR-199a-3p	RAD9A	hsa-miR-485-5p	RAD9A	hsa-miR-324-3p	STOML1
hsa-miR-34a-5p	RAD9A	hsa-miR-485-5p	RAD9A	hsa-miR-146b-5p	STOML1

We conducted a search in the CHIPBase database (version 3.0) to identify Transcription factors (TFS) that bind to 5 Co-DEGs, resulting in the identification of 3 Co-DEGs (AAMDC, GPR158, GPRS) after rigorous screening. The interaction data between RAD9A and 30 transcription factors (TFS) of 35 pairs of interaction data were visualized by Cytoscape software (**Figure 5D**). mRNA-TF interaction relationships are shown in **Table 5**.

**Table 5:** mRNA-TF interaction network nodes.

mRNA	TF	mRNA	TF	mRNA	TF
AAMDC	ARID2	AAMDC	SP2	RAD9A	BRD2
AAMDC	BRD1	AAMDC	STAG1	RAD9A	E2F1
AAMDC	HOXA4	AAMDC	TBP	RAD9A	HDAC1
AAMDC	IKZF1	AAMDC	TFAP2A	RAD9A	HIF1A
AAMDC	KLF4	AAMDC	TFAP2C	RAD9A	IRF1
AAMDC	LIN9	AAMDC	YY1	RAD9A	NFYA
AAMDC	MBD4	GPR158	GRHL2	RAD9A	NRF1
AAMDC	MYC	GPR158	REST	RAD9A	SP1
AAMDC	NFYA	GPR158	TFAP2A	RAD9A	TBP
AAMDC	POLR2A	GPR158	TFAP2C	RAD9A	TFDP1
AAMDC	SMAD3	GPR158	ZBTB7A	RAD9A	ZFX
AAMDC	SP1	RAD9A	ARID4B		

TF, Transcription factors.

We utilized the CTD database to predict potential drugs or small molecule compounds that interact with 5 Co-DEGs. Ultimately, we identified a total of 95 drug molecules composed of 4 Co-DEGs (AAMDC, GPR158, RAD9A, STOML1). Furthermore, this analysis resulted in the formation of 138 mRNA-drug interaction relationships (**Figure 5E**), which are detailed in **Table 6**.

**Table 6:** mRNA-drug interaction network nodes.

mRNA	Drug	mRNA	Drug
AAMDC	1-Methyl-3-isobutylxanthine	RAD9A	Aflatoxin B1
AAMDC	4-(4-(5-(4,5-dimethyl-2-nitrophenyl)-2-furanyl)methylene)-4,5-dihydro-3-methyl-5-oxo-1H-pyrazol-1-yl)benzoic acid	RAD9A	aristolochic acid I
AAMDC	4,4’-hexafluorisopropylidene diphenol	RAD9A	Arsenic Trioxide
AAMDC	7, 8-dihydro-7,8-dihydroxybenzo(a)pyrene 9,10-oxide	RAD9A	benzo(e)pyrene
AAMDC	Aflatoxin B1	RAD9A	bis(4-hydroxyphenyl)sulfone
AAMDC	Air Pollutants	RAD9A	bis-N,N-dimethylamino-2-(N-methylpyrrolyl)methyl cyclopentadienyl titanium (IV)
AAMDC	Arsenic	RAD9A	bisphenol A
AAMDC	arsenite	RAD9A	bromoacetate
AAMDC	Benzo(a)pyrene	RAD9A	Carmustine
AAMDC	bis(4-hydroxyphenyl)sulfone	RAD9A	Catechin
AAMDC	bisphenol A	RAD9A	coumarin
AAMDC	bisphenol F	RAD9A	CPG-oligonucleotide
AAMDC	Caffeine	RAD9A	deguelin
AAMDC	chromium hexavalent ion	RAD9A	Ethyl Methanesulfonate
AAMDC	Cyclosporine	RAD9A	Formaldehyde
AAMDC	Dexamethasone	RAD9A	Grape Seed Proanthocyanidins
AAMDC	dicrotophos	RAD9A	jinfukang
AAMDC	Doxorubicin	RAD9A	licochalcone B
AAMDC	epigallocatechin gallate	RAD9A	Methapyrilene
AAMDC	Estradiol	RAD9A	Methyl Methanesulfonate
AAMDC	Ethyl Methanesulfonate	RAD9A	methylselenic acid
AAMDC	Formaldehyde	RAD9A	myristicin
AAMDC	Gasoline	RAD9A	Nonidet P-40
AAMDC	Indomethacin	RAD9A	Resveratrol
AAMDC	Ivermectin	RAD9A	Rotenone
AAMDC	(+)-JQ1 compound	RAD9A	Smoke
AAMDC	Methyl Methanesulfonate	RAD9A	Sunitinib
AAMDC	Nickel	RAD9A	Taurine
AAMDC	Nitrogen Dioxide	RAD9A	Temozolomide
AAMDC	Particulate Matter	RAD9A	Theophylline
AAMDC	Plant Extracts	RAD9A	Tobacco Smoke Pollution
AAMDC	Polycyclic Aromatic Hydrocarbons	RAD9A	Tretinoin
AAMDC	potassium chromate(VI)	RAD9A	trichostatin A
AAMDC	Resveratrol	RAD9A	Tunicamycin
AAMDC	Silicon Dioxide	RAD9A	Valproic Acid
AAMDC	Sunitinib	STOML1	1-Methyl-3-isobutylxanthine
AAMDC	Tretinoin	STOML1	4-(4-(5-(4,5-dimethyl-2-nitrophenyl)-2-furanyl)methylene)-4,5-dihydro-3-methyl-5-oxo-1H-pyrazol-1-yl)benzoic acid
AAMDC	trichostatin A	STOML1	abrine
AAMDC	Tris (1, 3 - dichloro - 2 - propyl) phosphate	STOML1	Acetaminophen
AAMDC	Urethane	STOML1	Aflatoxin B1
AAMDC	Valproic Acid	STOML1	Antirheumatic Agents
AAMDC	Vincristine	STOML1	aristolochic acid I
AAMDC	Vorinostat	STOML1	Atrazine
GPR158	2,2’,4,4’-tetrabromodiphenyl ether	STOML1	Benzo(a)pyrene
GPR158	4 - (5 - benzo (1, 3) dioxol - 5 - yl - 4 - pyridin - 2 - h - imidazol - 2 yl - 1 - yl) benzamide	STOML1	benzo(e)pyrene
GPR158	abrine	STOML1	bisphenol A
GPR158	Aflatoxin B1	STOML1	bisphenol F
GPR158	aflatoxin B2	STOML1	Calcitriol
GPR158	arsenite	STOML1	Cannabidiol
GPR158	Benzo(a)pyrene	STOML1	Dexamethasone
GPR158	bis(4-hydroxyphenyl)sulfone	STOML1	Diethylhexyl Phthalate
GPR158	bisphenol A	STOML1	Formaldehyde
GPR158	Cadmium	STOML1	GSK-J4
GPR158	Cadmium Chloride	STOML1	Indomethacin
GPR158	Cyclosporine	STOML1	K 7174
GPR158	Dihydrotestosterone	STOML1	Lead
GPR158	dorsomorphin	STOML1	Methapyrilene
GPR158	Folic Acid	STOML1	methylmercuric chloride
GPR158	Fulvestrant	STOML1	Methyl Methanesulfonate
GPR158	Sunitinib	STOML1	Plant Extracts
GPR158	trichostatin A	STOML1	Resveratrol
GPR158	Valproic Acid	STOML1	Smoke
RAD9A	3-(2-hydroxy-4-(2-methylnonan-2-yl)phenyl)cyclohexan-1-ol	STOML1	sodium arsenite
RAD9A	4,4’-hexafluorisopropylidene diphenol	STOML1	Sunitinib
RAD9A	7, 3 ‘- dihydroxy - 4’ - methoxyisoflavone	STOML1	Tetrachlorodibenzodioxin
RAD9A	abrine	STOML1	Tobacco Smoke Pollution
RAD9A	Acetaminophen	STOML1	Valproic Acid
RAD9A	Acetylcysteine	STOML1	Vitamin E
RAD9A	Acrylamide	STOML1	Zoledronic Acid

### 3.5 GSEA between OE and NG groups

**Figure 6:**
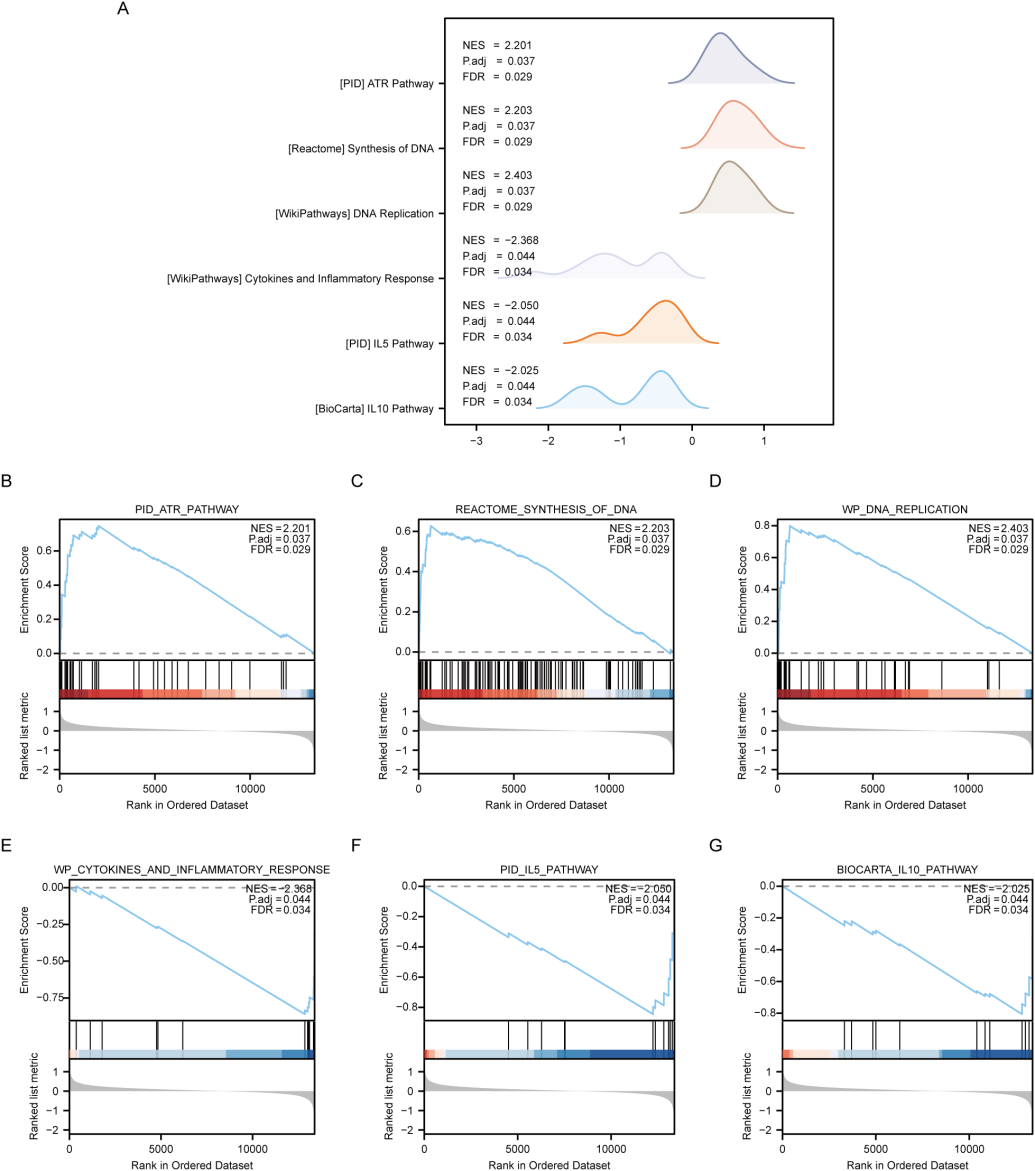
GSEA enrichment analysis between OE group and NG group. **(A)**. Six main biological features of GSEA enrichment analysis between OE and NG groups. B-g. Genes significantly enriched in PID_ATR_PATHWAY **(B)**, REACTOME_SYNTHESIS_OF_DNA **(C)**, WP_DNA_REPLICATION **(D)** between OE group and NG group. WP_CYTOKINES_AND_INFLAMMATORY_RESPONSE **(E)**, PID_IL5_PATHWAY **(F)**, BIOCARTA_IL10_PATHWAY **(G)**. OE, Overexpression group; NG, Normal group; GSEA, Gene Set Enrichment Analysis. The significant enrichment screening criteria for GSEA enrichment analysis were p. Adj < 0.05 and FDR value (q.value) < 0.05.

The Gene Set Enrichment Analysis (GSEA) was employed to analyze the relationship between gene expression and biological processes, cellular components and molecular functions of OE and NG groups. Significance enrichment was determined based on p.adj < 0.05 and FDR value (q.value) < 0.05 criteria. The results showed that genes in the Combined dataset disease Control group (CD/Control) were significantly enriched in PID_ATR_PATHWAY (**Figure 6B**), REACTOME_SYNTHESIS_OF_DNA (**Figure 6C**), and P. WP_DNA_REPLICATION (**Figure 6D**), WP_CYTOKINES_AND_INFLAMMATORY_RESPONSE (**Figure 6E**), PID_IL5_PATHWAY (**Figure 6F**), BIOCARTA_IL10_PATHWAY (**Figure 6G**) and other pathways (see **Table 7** for pathway details). Furthermore, a mountain plot was utilized to present the results of GSEA for genes between OE and NG groups. (**Figure 6A**).

**Table 7:** Result of GSEA enrichment analysis.

ID	setSize	enrichmentScore	NES	p.adjust	*P*
BIOCARTA_IL10_PATHWAY	13	0.80602	2.02495	0.044149	0.034491
PID_IL5_PATHWAY	12	0.84514	2.04976	0.044149	0.034491
WP_CYTOKINES_AND_INFLAMMATORY_RESPONSE	17	0.86001	2.36777	0.044149	0.034491
WP_DNA_REPLICATION	42	0.799286	2.403438	0.037135	0.029011
REACTOME_SYNTHESIS_OF_DNA	118	0.627898	2.203185	0.037135	0.029011
PID_ATR_PATHWAY	37	0.747404	2.201419	0.037135	0.029011

GSEA, Gene Set Enrichment Analysis.

### 3.6 GSVA between OE group and NG group

**Figure 7:**
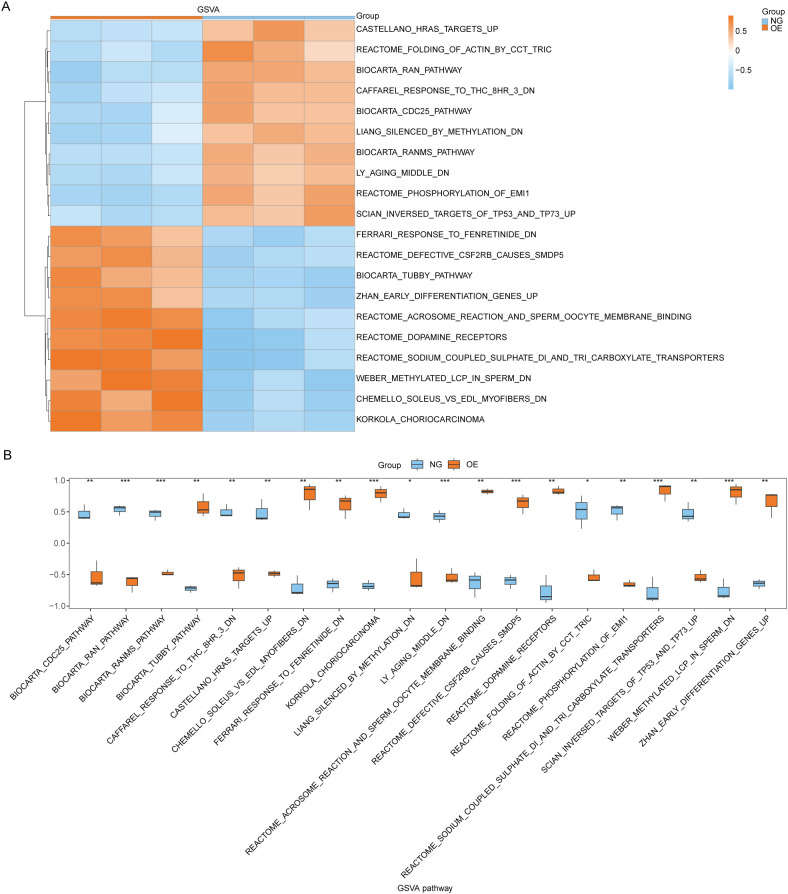
GSVA enrichment analysis between OE group and NG group. **(A, B)**. Complex numerical heat map of GSVA enrichment analysis results between OE group and NG group **(A)**, group comparison plot display **(B)**. OE, Overexpression group; NG, Normal group; GSVA: Gene Set Variation Analysis. The symbol ns is equivalent to *P* ≥ 0.05 and has no statistical significance; The symbol * is equivalent to *P* < 0.05, which is statistically significant; The symbol ** is equivalent to *P* < 0.01, which is highly statistically significant; The symbol *** is equivalent to *P* < 0.001 and highly statistically significant.

Subsequently, we conducted Gene Set Variation Analysis (GSVA) by selecting the top 10 pathways with the largest and smallest logFC values from pathways with P<0.05 for further analysis (refer to **Table 8** for details). The GSVA analysis of all genes between OE group and NG group revealed significant differences in a total of 20 pathways including methylated_lcp_in_sperm, soleus_vs_edl_myofibers, and korkola_choriocarcinoma. Based on these results, we performed differential expression analysis of these 20 pathways between the two groups and visualized them using pheatmap R package to generate a heatmap illustrating specific differential patterns (**Figure 7A**). Additionally, we assessed the degree of group differences in these 20 pathways between the OE group and the NG group through comparative mapping (**Figure 7B**), which demonstrated statistically significant distinctions in their expression levels (P<0.05).

**Table 8:** Result of GSVA enrichment analysis.

	logFC	AveExpr	t	*P*
REACTOME_PHOSPHORYLATION_OF_EMI1	1.16234	0.07139	5.77164	0.000522
BIOCARTA_RAN_PATHWAY	1.16218	0.04646	5.82187	0.000494
REACTOME_FOLDING_OF_ACTIN_BY_CCT_TRIC	1.04244	0.01314	4.83501	0.001536
CAFFAREL_RESPONSE_TO_THC_8HR_3_DN	1.02418	0.01333	5.12825	0.001081
SCIAN_INVERSED_TARGETS_OF_TP53_AND_TP73_UP	1.01415	0.03147	5.04746	0.00119
LIANG_SILENCED_BY_METHYLATION_DN	0.99671	0.04186	4.55134	0.002181
BIOCARTA_CDC25_PATHWAY	0.99666	0.02864	4.68778	0.00184
CASTELLANO_HRAS_TARGETS_UP	0.97644	0.002064	5.05819	0.001175
LY_AGING_MIDDLE_DN	0.96479	0.05538	4.7962	0.00161
BIOCARTA_RANMS_PATHWAY	0.9373	0.00649	5.11737	0.001095
REACTOME_DEFECTIVE_CSF2RB_CAUSES_SMDP5	1.241498	0.015158	6.556207	0.000231
FERRARI_RESPONSE_TO_FENRETINIDE_DN	1.267578	0.02803	6.130772	0.000356
ZHAN_EARLY_DIFFERENTIATION_GENES_UP	1.294987	0.001783	6.466227	0.000252
BIOCARTA_TUBBY_PATHWAY	1.309154	0.07036	6.260685	0.000311
REACTOME_ACROSOME_REACTION_AND_SPERM_OOCYTE_MEMBRANE_BINDING	1.461783	0.092536	8.844684	3.06 e-05
KORKOLA_CHORIOCARCINOMA	1.465057	0.054778	8.683449	3.47 e-05
CHEMELLO_SOLEUS_VS_EDL_MYOFIBERS_DN	1.478569	0.035895	7.384433	0.000105
WEBER_METHYLATED_LCP_IN_SPERM_DN	1.561074	0.022535	7.962227	6.30 e-05
REACTOME_DOPAMINE_RECEPTORS	1.602557	0.032032	8.262094	4.89 e-05
REACTOME_SODIUM_COUPLED_SULPHATE_DI_AND_TRI_CARBOXYLATE_TRANSPORTERS	1.606512	0.026145	8.082214	5.69 e-05

GSVA, Gene Set Variation Analysis.

### 3.7 Difference analysis of ssGSEA immune characteristics between OE group and NG group

**Figure 8:**
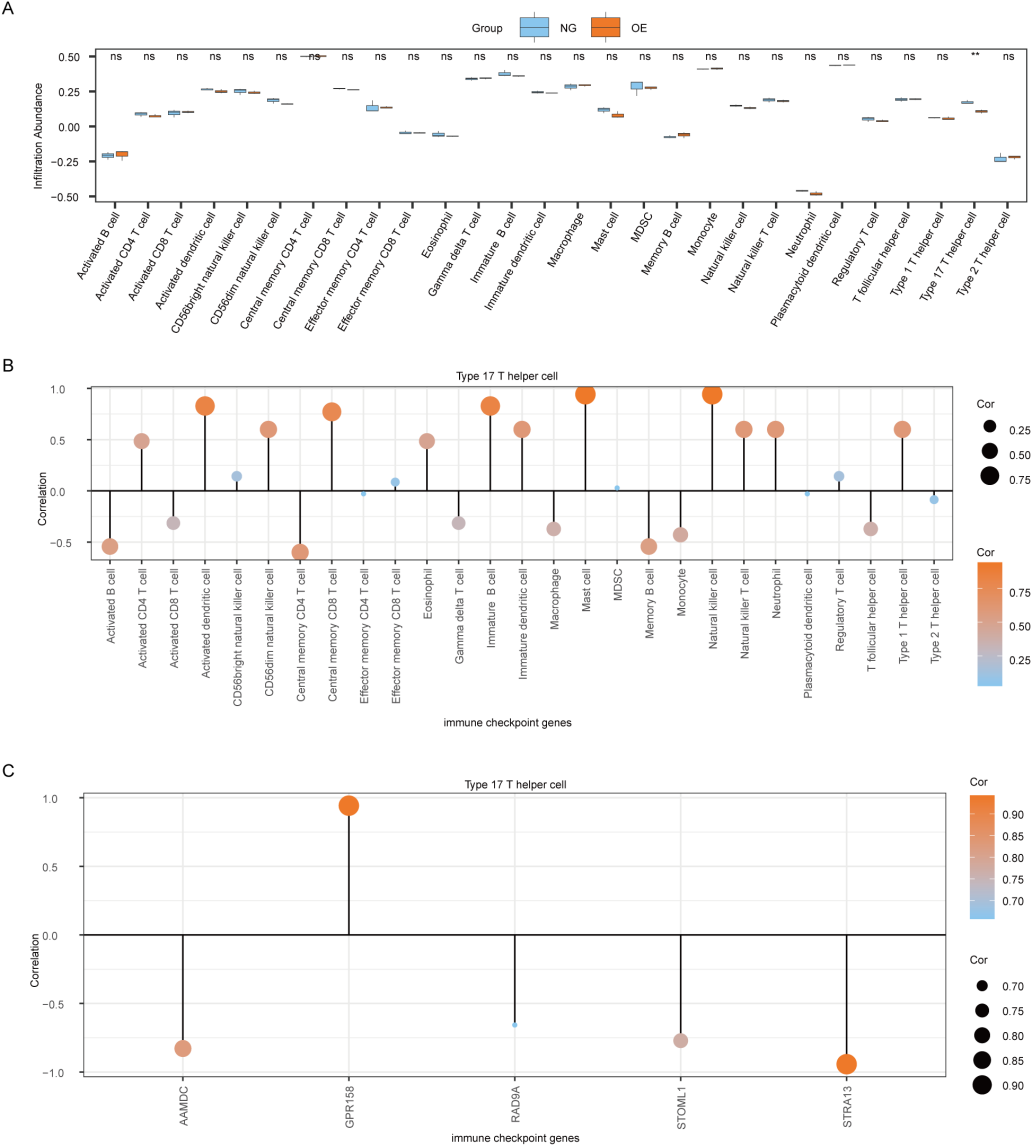
Analysis of ssGSEA immune characteristics between OE group and NG group. **(A)**. Group comparison of ssGSEA immune infiltration analysis results between OE group and NG group is shown. **(B)**. Lollipop chart showing the correlation between Type 17 T helper cells and immune cells. **(C)**. Lollipop diagram of the correlation between Type 17 T helper cells and Co-DEGs. The symbol ns is equivalent to P≥ 0.05, which is not statistically significant. The symbol ** is equivalent to *P* < 0.01, which is highly statistically significant; and highly statistically significant. OE, Overexpression group; NG, Normal group; ssGSEA, single-sample gene-set enrichment Analysis; Co-DEGs, Common differentially expressed genes.

To explore the difference in immune infiltration between the OE group and the NG group, we used the ssGSEA algorithm to calculate the infiltration abundance of 28 immune cells in the OE group and the NG group samples, and then displayed the difference degree of infiltration of 28 immune cells by group comparison plot (**Figure 8A**). The results showed that the infiltration abundance of Type 17 T helper cells was significantly different between the OE group and the NG group (*P*<0.05).

Then we plotted the correlation lollipop diagram to show the correlation between Type 17 T helper cells and other immune cells. According to the figure, Mast cells and Natural killer cells were the most strongly correlated with Type 17 T helper cells (**Figure 8B**).

At the same time, we also plotted the correlation lollipop graph to show the correlation between Type 17 T helper cells and 5 Co-DEGs (AAMDC, GPR158, RAD9A, STOML1, STRA13). The results showed that Type 17 T helper cells were strongly correlated with GPR158 and STRA13 (**Figure 8C**).

### 3.8 CIBERSORT immunosignature difference analysis between OE group and NG group

**Figure 9:**
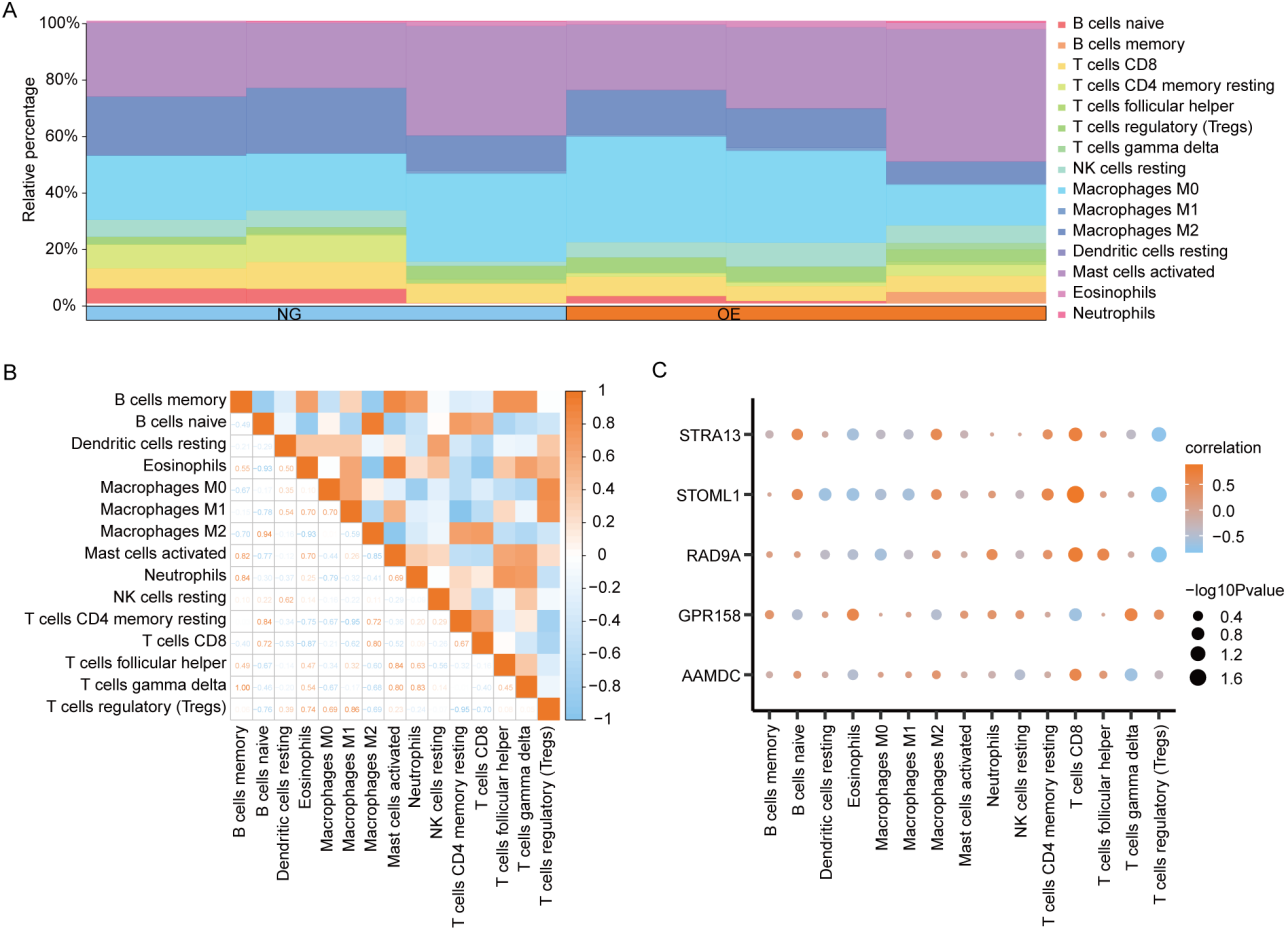
CIBERSORT immune characteristics difference analysis between OE group and NG group. **(A)**. The infiltration abundance of immune cells between OE group and NG group was shown by stacking bar chart. **(B)**. The correlation between immune cells is shown. **(C)**. Dot plot of correlation between immune cells and Co-DEGs. OE, Overexpression group; NG, Normal group; Co-DEGs, Common differentially expressed genes.

The CIBERSORT algorithm was employed to calculate the infiltration abundance of 22 immune cells in the disease Control group (CD/Control). A stacked bar chart was utilized to visually represent the proportion of immune cells visually represent (**Figure 9A**). The findings revealed that the infiltrating abundance of 15 immune cells in the Combined dataset samples was not all 0, and these 15 immune cells were B cells memory, B cells naive, Dendritic cells resting, Eosinophils, and eosinophils. Macrophages M0, Macrophages M1, Macrophages M2, Mast cells activated, Neutrophils, NK cells resting, T cells CD4 memory resting, T cells CD8, T follicular helper (Tfh)cells, γδT-cells and regulatory T-cells (Tregs).

The spearman statistical algorithm was employed to compute the correlation among 15distinct immune cell types, revealing a balanced distribution of positive and negative correlations. Notably, Macrophages M2 and B cells naive exhibited the most robust association.

We subsequently employed Spearman’s statistical algorithm to calculate the correlation between 15 immune cells and 5 Co-DEGs (AAMDC, GPR158, RAD9A, STOML1, STRA13) (**Figure 9B**). The findings revealed a balanced distribution of positive and negative correlations among the immune cells and Co-DEGs. Notably, T cells CD8 exhibited the strongest association with STOML1 (**Figure 9C**).

### 3.9 Validation of differentially expressed gene results

**Figure 10:**
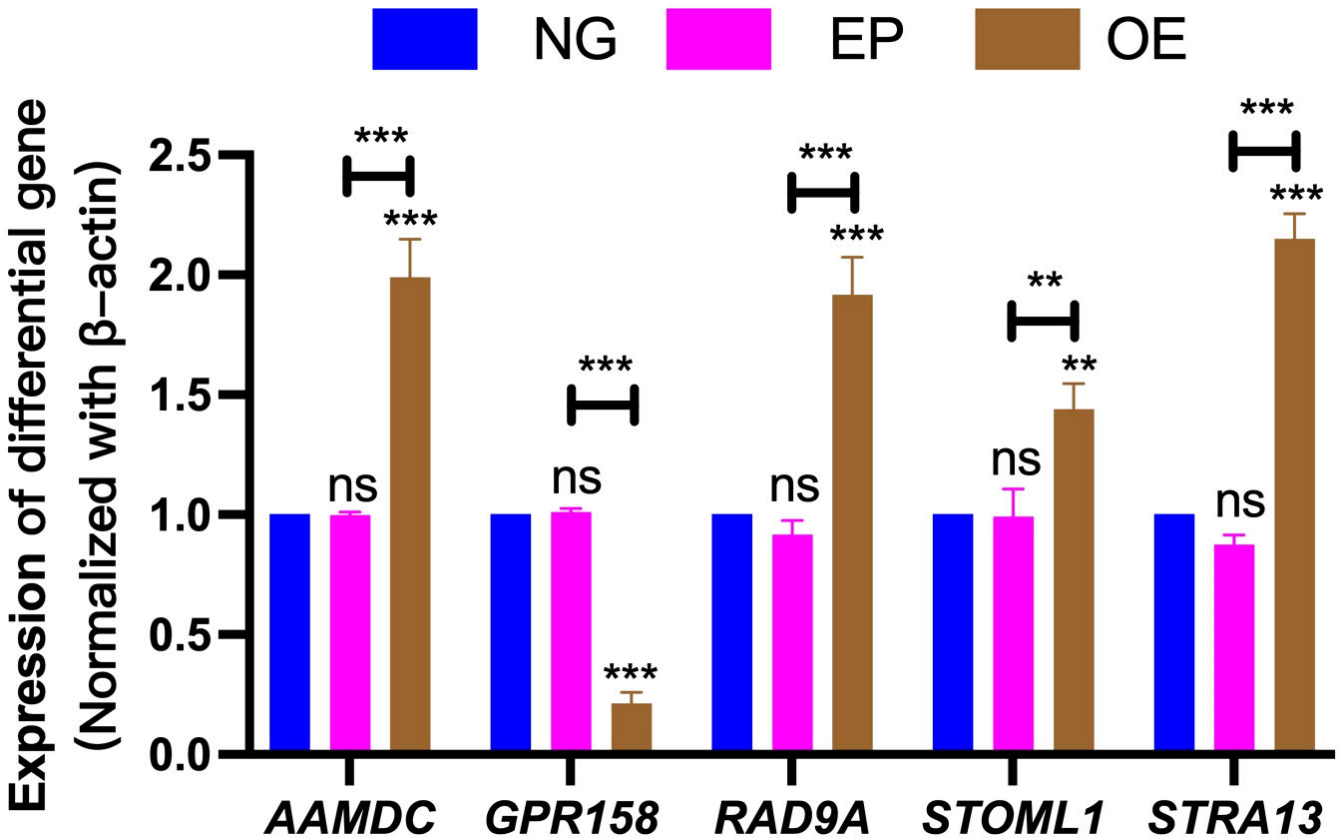
Validation of differentially expressed gene results. Verification of differentially expressed genes AAMDC, GPR158, RAD9A, STOML1 and STRA13 among NG, EP and OE groups. NG, Normal group;EP, Empty group; OE, Overexpression group.The symbol ns was equivalent to *P* ≥ 0.05, which was not statistically significant. The symbol ** is equivalent to *P* < 0.01, which is highly statistically significant; The symbol *** is equivalent to *P* < 0.001 and highly statistically significant.

The differential gene expression results were validated by performing RT-PCR analysis on five selected genes (AAMDC, GPR158, RAD9A, STOML1, and STRA13). These genes exhibited consistent patterns with the transcriptome analysis data; however, slight variations in individual values were observed due to disparities in method sensitivity (**Figure 10**).

## 4 Discussion

*T.gondii* has the ability to infect a wide range of warm-blooded animals, including humans (Bao et al., 2021). During the infection process of host nucleated cells by *T.gondii*, the ROP16 protein is secreted and released into the cytoplasmic lysate. It then rapidly translocates and localizes to the host nucleus by nuclear translocation sequences and exerts its function (Xin et al., 2020). In macrophages, which are an important preferential target cell for parasite infection, ROP16 plays diverse roles such as reducing inflammatory progenitor cytokines and macrophage differentiation (Jin et al., 2023).

The study of *T.gondii* infection necessitates an examination of immunity to infection, wherein macrophages assume a pivotal role owing to their ubiquitous presence in various tissues or organs, ability to migrate into tissues or organs during homeostatic or inflammatory states, and crucial defense function in the intrinsic immune response (Chen et al., 2020).

Because macrophages are crucial for studying the immune mechanism of infection (Jiménez-Garcia et al., 2015), the direct use of primary macrophages in experiments closely mimics the physiological state compared to cell lines. However, due to challenges associated with obtaining primary macrophages, individual differences, individual variations, labor-intensive procedures, and limited survival time during isolation, stable transmissible rat or mouse macrophage cell lines or induced monocytes as macrophages are still the main source of macrophages in current experimental research. The present study aimed to investigate the direct or indirect transcriptional regulation and heterogeneous effects of *T.gondii* ROP16 II on human-derived THP-1 macrophages, differentiated by PMA (light stabilizer 12-mystate 13-acetate), which holds significant research implications (Jiménez-Garcia et al., 2015; Yoo et al., 2022). Additionally, we sought to explore the regulation of genes and signaling pathways associated with human-derived macrophages following *rop16* overexpression, potentially offering a novel immunotherapy strategy utilizing effector molecules derived from parasites.

The aim of this study was to investigate the relationship between toxoplasmosis and target genes. This was achieved by conducting differential expression analysis to screen Co-DEGs, followed by GO, GSEA and GSVA analysis. Additionally, a PPI network and mRNA interaction network were constructed to elucidate the interplay among these genes. Furthermore, immune infiltration analysis was performed using the ssGSEA algorithm.

The present study employed bioinformatics analysis to identify five key genes, namely AAMDC, GPR158, RAD9A, STOML1, and STRA13 among the Co-DEGs. These genes are potentially involved in the regulatory mechanism of *T.gondii* ROP16 protein-infected cells and may affect the occurrence and prognosis of toxoplasmosis through the regulation of infection. Furthermore, the expression patterns of these genes are associated with the onset, progression, and metastasis of toxoplasmosis. They also hold promise as potential therapeutic targets for developing new treatments against toxoplasmosis. Additionally, we observed significant differences in 20 pathways related to hras targets, folding of actin by cct tric, and ran pathway among CD/Control datasets for disease controls. Notably enriched pathways included atr pathway, synthesis of DNA, DNA replication, cytokines and inflammatory response, IL5 pathway as well as L10 pathway. Moreover, the abundance of Type 17 T helper cell infiltration showed statistically significantly different between the OE and NG groups (*P*<0.05). By analyzing mRNA-miRNA, mRNA-RBP, and mRNA-TF interactions networks, it is possible to gain insights into the regulatory mechanisms underlying toxoplasmosis-related gene expression. Furthermore, this analysis can help identify potential regulatory pathways involved in this process. Immune cell infiltration analysis revealed strong correlations between Mast cell, Natural killer cell and Type 17 T helper cell. Type 17 T helper cell exhibited a strong correlation with GPR158 and STRA13. Among immune cell correlations the strongest correlation was between M2 Macrophages and B cells naive, and cellular CD8 T cells correlated most strongly with STOML1.

The key gene AAMDC plays a crucial role in cellular energy utilization and storage by interacting with multiple signaling pathways. Their specific structural domains are essential for regulating cell differentiation, metabolism and formation (Xiao et al., 2019). In certain types of cancers (Golden et al., 2021), AAMDC proteins may have either promotional or inhibitory effects. GPR158 transduces signals through coupling to RGS proteins (Laboute et al., 2023) and regulates key ion channels, kinases, and neurotrophic factors involved in neuronal excitability and synaptic transmission by modulating signaling of the second messenger 3’,5’ -adenosine monophosphate (cAMP) (Piaggi et al., 2017). RAD9A is a cell cycle checkpoint protein required for cell cycle arrest and DNA damage repair. Its 3’ to 5’ nuclease activity may be related to its role in sensing and repairing DNA damage (Vasileva et al., 2013; Pelaia et al., 2016). Stomatin Like 1 (STOML1) is a protein-coding gene highly expressed in the brain but with lower expression levels observed in cardiac, skeletal muscle, and DRG sensory neurons (Kozlenkov et al., 2014). Significantly levels of STOML 1 expression are detected in the frontal lobe, cerebral cortex, hippocampus, and other basal ganglia. STOML1 consists of a structural domain resembling stomatocystin and SCP -2, an acid-sensitive cell membrane protein that regulates acid sensitivity in ion channels, and is believed to regulate the function of ion channels and transporter proteins (Edqvist and Blomqvist, 2006; Wang et al., 2020). STRA13 is a transcription factor involved in immune cell homeostasis regulation autoimmunity; it is up-regulated at mRNA levels across various cancer cell lines (Ivanova et al., 2004). These genes have been shown to participate in processes such as cell differentiation and regeneration, circadian regulation, immune homeostasis and metabolism (Ow et al., 2014). The identification of AAMDC, GPR158, RAD9A, STOML1, STRA13 genes provides potential molecular therapeutic or immunotherapeutic targets that could contribute towards precision diagnosis and personalized treatment for *T.gondii* infection breakthroughs.

In light of these crucial factors and in conjunction with the present study, it is of immense significance to comprehend the modulation of host immune function by *T.gondii* ROP16 protein, elucidate the inter-regulatory mechanism between ROP16 and host macrophages, unravel the underlying mechanism governing the transition between pro-inflammatory and anti-inflammatory roles of immune cells, as well as to identify novel targets for drug intervention in immune-inflammation.

The pathways significantly enriched in this study included the ATR pathway, DNA synthesis, DNA replication, cytokines and inflammatory response, IL5 pathway, and L10 pathway. Among these pathways IL-5 primarily produced by T cells, acts as the key cytokine influencing eosinophil growth, differentiation, recruitment, activation, and survival. It also impacts the activation of several subsequent signaling pathways including JAK/STAT, MAPK, PI3K, and NF-κB, which are responsible for transcription of genes involved in eosinophil differentiation, degranulation, survival, proliferation, chemotaxis, and adhesion (Takatsu and Nakajima, 2008). Interleukin 10 (IL-10), a pleiotropic cytokine with potent anti-inflammatory properties, is mainly secreted by antigen-presenting cells such as activated T cells, monocytes, B cells, and macrophages. It inhibits the expression of inflammatory cytokines, such as TNF-α, IL-6, and IL-1 by activating macrophages (Pelaia et al., 2016). The enrichment of these pathways aligns with the findings of this study suggesting that *rop16* overexpression indeed affects host macrophage-associated inflammatory factors secretion and expression.

This study has several limitations. Firstly, the bioinformatics analysis results were solely based on data obtained from *rop16* overexpression in cell lines without further functional validation through wet experiments. Secondly, the lack of corresponding clinical correlation studies prevented the analysis of clinical information alongside the findings. Lastly, potential batch-to-batch variations within the large dataset or insufficient sample sizes may affected the reliability and stability of the experiment’s; thus, a larger sample size is required for ensuring robustness. Consequently, this paper provides a comprehensively exploration of toxoplasmosis pathogenesis and presents a scoring model. Nevertheless, further verification is necessary to elucidate specific pathogenic and molecular targets.

## 5 Conclusion

The pivotal genes AAMDC, GPR158, RAD9A, STOML1and STRA13 identified in this study have the potential to serve as molecular therapeutic or immunotherapeutic targets for toxoplasmosis; however, their specific function mechanisms require further verification. Additionally, the high expression of one of the AAMDC genes suggests a possible avenue for using toxoplasma proteins to treat tumors. These findings enhance our understanding of ROP16 and may inform future therapeutic strategies against toxoplasmosis by guiding the development of new and safer approaches.

## Data Availability

The original contributions presented in the study are included in the article/supplementary material. Further inquiries can be directed to the corresponding author.
